# Engagement of Nuclear Coactivator 7 by 3-Hydroxyanthranilic Acid Enhances Activation of Aryl Hydrocarbon Receptor in Immunoregulatory Dendritic Cells

**DOI:** 10.3389/fimmu.2019.01973

**Published:** 2019-08-20

**Authors:** Marco Gargaro, Carmine Vacca, Serena Massari, Giulia Scalisi, Giorgia Manni, Giada Mondanelli, Emilia M. C. Mazza, Silvio Bicciato, Maria T. Pallotta, Ciriana Orabona, Maria L. Belladonna, Claudia Volpi, Roberta Bianchi, Davide Matino, Alberta Iacono, Eleonora Panfili, Elisa Proietti, Ioana Maria Iamandii, Violetta Cecchetti, Paolo Puccetti, Oriana Tabarrini, Francesca Fallarino, Ursula Grohmann

**Affiliations:** ^1^Department of Experimental Medicine, University of Perugia, Perugia, Italy; ^2^Department of Pharmaceutical Sciences, University of Perugia, Perugia, Italy; ^3^Laboratory of Translational Immunology, Istituto Clinico Humanitas IRCCS, Rozzano, Italy; ^4^Department of Biomedical Sciences, University of Modena and Reggio Emilia, Modena, Italy

**Keywords:** aryl hydrocarbon (Ah) receptor, nuclear coactivator 7 (NCOA7), tryptophan metabolism, dendritic cell, immune regulation

## Abstract

Indoleamine 2,3-dioxygenase 1 (IDO1) catalyzes the first step in the kynurenine pathway of tryptophan (Trp) degradation that produces several biologically active Trp metabolites. L-kynurenine (Kyn), the first byproduct by IDO1, promotes immunoregulatory effects via activation of the Aryl hydrocarbon Receptor (AhR) in dendritic cells (DCs) and T lymphocytes. We here identified the nuclear coactivator 7 (NCOA7) as a molecular target of 3-hydroxyanthranilic acid (3-HAA), a Trp metabolite produced downstream of Kyn along the kynurenine pathway. In cells overexpressing NCOA7 and AhR, the presence of 3-HAA increased the association of the two molecules and enhanced Kyn-driven, AhR-dependent gene transcription. Physiologically, conventional (cDCs) but not plasmacytoid DCs or other immune cells expressed high levels of NCOA7. In cocultures of CD4^+^ T cells with cDCs, the co-addition of Kyn and 3-HAA significantly increased the induction of Foxp3^+^ regulatory T cells and the production of immunosuppressive transforming growth factor β in an NCOA7-dependent fashion. Thus, the co-presence of NCOA7 and the Trp metabolite 3-HAA can selectively enhance the activation of ubiquitary AhR in cDCs and consequent immunoregulatory effects. Because NCOA7 is often overexpressed and/or mutated in tumor microenvironments, our current data may provide evidence for a new immune check-point mechanism based on Trp metabolism and AhR.

## Introduction

Immune regulation is a highly evolved form of biologic response that controls immunity but also dampens exaggerated inflammation (1–4). Indoleamine 2,3-dioxygenase 1 (IDO1) and the Aryl hydrocarbon Receptor (AhR) represent two ancient metabolic molecules that may have functionally co-evolved to integrate their immunoregulatory potential (5, 6).

IDO1 is an inducible, rate-limiting enzyme in the kynurenine pathway of l-tryptophan (Trp) metabolism generating l-kynurenine (Kyn) and multiple downstream metabolites, collectively termed kynurenines, which include 3-hydroxykynurenine, 3-hydroxyanthranilic acid (3-HAA), and quinolinic acid (Figure S1) (7, 8). Among immune cells, dendritic cells (DCs) express the highest levels of IDO1, particularly in response to the cytokine interferon γ (IFN-γ) (9–11). Trp starvation and the production of kynurenines by DCs induce the conversion of naïve CD4^+^ T cells into Foxp3^+^ regulatory T (Treg) cells (12, 13). Nevertheless, the high levels of Kyn produced by DCs under inflammatory conditions—as well as by tumors—can exert immunoregulatory effects on T cells via AhR activation in the absence of Trp starvation (5, 14).

Among the Trp metabolites produced downstream of Kyn along the kynurenine pathway, 3-HAA appears to be highly relevant in immune regulation. 3-HAA has been indeed shown to be a biological mediator against inflammation and allograft rejection via inhibition of effector responses or induction of apoptosis in T cells (15–19). Moreover, 3-HAA enhances the immunosuppressive effects of IDO1-expressing DCs in an allograft model of small bowel transplantation, favoring expansion of Treg cells (20). Oral administration of a synthetic derivative of 3-HAA protects mice from the development of experimental autoimmune encephalomyelitis, an experimental model of human multiple sclerosis (21). The concept of 3-HAA as an immunoregulatory molecule is also consistent with studies showing significant decrease in its plasma levels in inflammatory disorders as well as their normalization after therapeutic treatment (22). Previous evidence indicates that 3-HAA is involved in the activation of PDK1 kinase (16) and caspase 8 (19) in mediating T-cell apoptosis. Moreover, 3-HAA has been shown to activate AhR-mediated effects in T cells, although it is cinnabarinic acid—a dimerized form of 3-HAA produced in oxidant conditions—rather than 3-HAA, that binds and activates AhR (23). As a whole, the available data would indicate that the precise nature of the molecular target/s of 3-HAA in immune regulation is/are still unclear.

AhR is a transcription factor activated by several exogenous and endogenous ligands (24). Initially recognized as the receptor mediating the toxic effects of 2,3,7,8-tetrachlorodibenzo-*p*-dioxin (TCDD), AhR is now considered as an important modulator of cell physiology and organ homeostasis (25). AhR expression is essentially ubiquitous in mammals, compatible with its broad spectrum of activity (26). However, the nature of the ligand as well as that of the tissue, which may provide a specific set of coactivators, may dictate the functional outcome of AhR activation (5). Among several physiologic effects, AhR contributes to immune homeostasis (24, 27, 28), by promoting immunoregulatory and host-protective effects (29, 30). In the control of immune responses, IDO1 and AhR have been found to be functionally interconnected (6). Kyn represents a major endogenous ligand in the activation of immunoregulatory AhR (31), yet activated AhR can modulate IDO1 expression in DCs (29, 32) and tumor cells (33). Besides Kyn, no other Trp metabolite in the kynurenine pathway appears to play a significant role in the functional circuitry established by AhR and IDO1 in immune regulation.

In the present study, by means of a pull-down methodology with a biotinylated derivative, we identified nuclear coactivator 7 (NCOA7) as a main target for 3-HAA. Moreover, we found that (i) NCOA7 is highly expressed in DCs; (ii) NCOA7 engagement by 3-HAA favors its association with AhR and increases Kyn-driven transcriptional activity of AhR; and (iii) the combined effects of 3-HAA and Kyn on NCOA7 and AhR, respectively, are necessary for the full empowerment of DCs in the induction of Treg cells.

## Materials and Methods

### Generation of Biotin–Tagged 3-HAA

**Figure 1:**
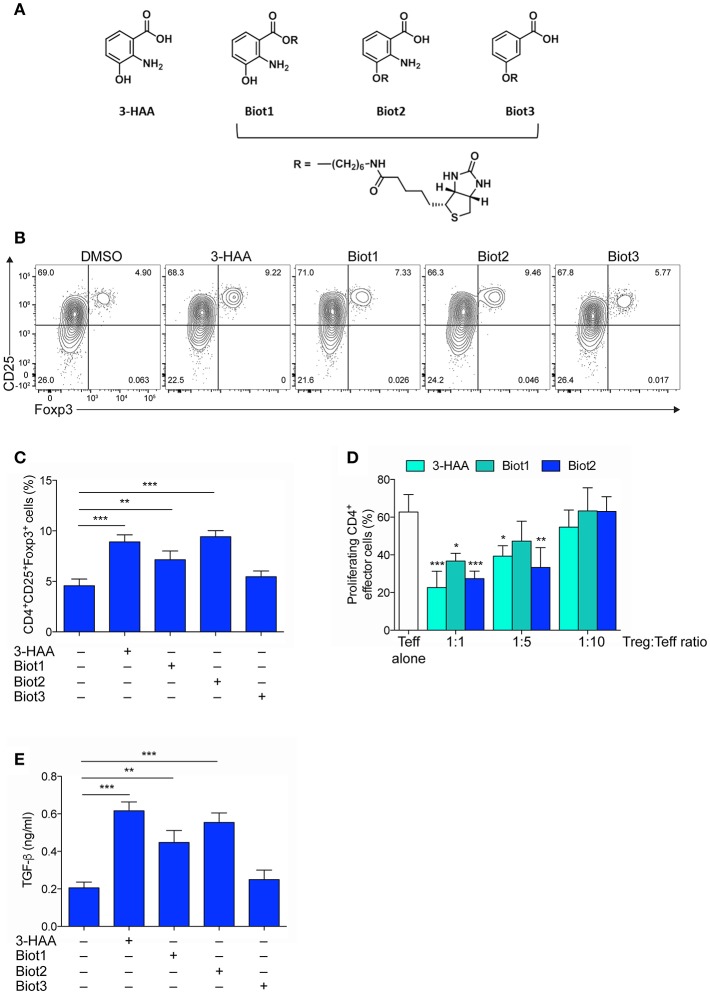
Selective Biotinylation Does Not Impair 3-HAA Immunoregulatory Effects. **(A)** Chemical Structures of 3-HAA and its biotinylated derivatives. **(B)** Representative dot plots of CD25 and Foxp3 coexpression in CD4^+^ cells that was evaluated by cytofluorometry in whole MLN cells after activation with anti-CD3 and -CD28 antibodies, in the presence or absence of 50 μM of 3-HAA, Biot1, Biot2, Biot3, or medium alone (control) for 72 h. A mouse IgG2a antibody was used as isotype control. Shown in upper right quadrant are percentages of double-positive cells. One experiment representative of three, whose means are shown in **(C)**. **(D)** Treg-mediated suppression as measured by CFSE dilution. CD4^+^CD25^−^ effector T cells (Teff) were purified from mouse MLN, labeled with 5 μM CFSE, activated with anti-CD3 and anti-CD28 antibodies, and cultured alone or in the presence of CD4^+^Foxp3^+^ cells purified from the 48 h cultures as in **(B)**, at different Teff:Treg ratios. After 72 h, proliferation was determined by cytofluorimetric analysis. One experiment representative of three. **(E)** TGF-β levels in supernatants of cultures established for 24 h as in **(A)**. DMSO, vehicle control. ^*^p < 0.05; ^**^p < 0.01; ^***^p < 0.001; (ANOVA followed by *post-hoc* Bonferroni's test).

No information about the functional groups of 3-HAA involved in the target recognition has been available so far. Therefore, we planned to synthesize three different compounds, by means of biotinylating alternatively the carboxylic, the amino, or the hydroxyl group of 3-HAA (Figure 1A). However, although we were able to synthesize Biot1 and Biot2 compounds (biotinylated in the carboxyl and hydroxyl group, respectively), synthetic difficulties hampered the insertion of the biotin moiety at the amino position. Because the introduction of a biotinyl group can alter the ligand structure decreasing its affinity (34), a hexamethylenic unit (i.e., C6) was inserted as a spacer arm (Figure 1A and Figure S2) between the ligand and the biotin molecule, to minimize steric hindrance. The biotin molecule was linked to the spacer through a classic amide bond and the spacer was coupled with the ligand through ester, as in Biot1, or an ether, as in Biot2 (Supplementary Methods). Biot3 was instead generated by tagging the hydroxyl group of 3-hydroxybenzoic acid, a compound structurally similar to 3-HAA but lacking the amino group (Supplementary Methods). All reactions were routinely checked by thin layer chromatography (TLC) on silica gel 60F254 (Merck) and visualized by using UV or iodine. Flash column chromatography separations were carried out on Merck silica gel 60 (mesh 230–400). Melting points were determined in capillary tubes (Büchi Electrothermal Mod. 9100) and were uncorrected. The purity of compounds was determined by HPLC Waters LC Module I Plus as the area of each peak and evaluated to be >97%. Purity was revealed at 228 nm (at λ max of each compounds) using a XTerra MS C18 column reversed-phase (3.5 μm spherical hybrid, 4.6 × 150 mm, 3.5 μm particle size) with 10 min at 1.0 ml/min isocratic of 40% acetonitrile (channel A) in 60% water with 0.1% formic acid (channel B). Injection volume was of 15 μL and column temperature of 30°C. ^1^H nuclear magnetic resonance (^1^H NMR) and ^13^C NMR spectra were recorded at 200 MHz (Bruker Avance DPX-200) and 400 MHz (Bruker Avance DRX-400) using residual solvents such as chloroform (CHCl_3_, δ = 7.26) or dimethylsulfoxide (DMSO; δ = 2.48) as an internal standard. Chemical shifts, given in ppm (δ), and spectral data were consistent with the assigned structures. Reagents and solvents were purchased from common commercial suppliers and were used as such. After extraction, organic solutions were dried over anhydrous Na_2_SO_4_, filtered, and concentrated with a Büchi rotary evaporator at reduced pressure. Yields are of purified product and were not optimized. All starting materials were commercially available unless otherwise indicated.

### Mice and Purification and Treatment of DCs and CD4^+^ T Cells

Eight- to ten-week-old female C57BL/6 mice were obtained from Charles River Breeding Laboratories. To evaluate the biologic activity of biotinylated compounds, total mesenteric lymph node (MLN) cells (0.2 × 10^6^) were incubated with soluble CD3-specific antibodies in the presence or absence of 3-HAA, Biot1, Biot2, or Biot3, at various concentrations as indicated. For the generation of conventional DCs (cDCs), bone marrow (BM) was harvested from the femur and tibia of mice. Bones were collected and fragmented by mortar and pestle in MACS buffer, and debris was removed by passing cells through a 70-μm strainer. Red blood cells were lysed with ACK lysis buffer and cells were subsequently counted. Bulk BM cells were cultured at 37°C in 4 ml total volume of complete IMDM supplemented with 100 ng/mL Flt3L (Peprotech) for 9 days before further analysis. Plasmacytoid DCs (pDCs) were depleted using B220 and CD317 magnetic conjugated beads. Resulting cDCs were >90% CD11c^+^ and >90% MHC I-A^+^/I-E^+^ (35, 36). CD4^+^CD25^−^ T cells were purified from MLNs as previously described (37, 38). In T cell–DC cocultures, 2 × 10^5^ CD4^+^CD25^−^ T cells were incubated at a 4:1 ratio with 0.5 × 10^5^ DCs, in the presence or absence of Kyn and/or 3-HAA, both at 50 μM, for 72 h in the presence of 2.5 μg/ml soluble antibody to CD3 (145-2C11, BD Pharmingen), as previously described (37, 38). For silencing of *Ncoa7*, gene-specific small interfering RNA (siRNA) was predesigned on the basis of the gene sequence and was synthesized by Ambion Life Technologies, which also supplied the negative control siRNA. Gene silencing in cDCs and MLN cells was as described (39). All experiments involving mice have been approved by Istituto Superiore di Sanità, Ministry of Health, Italy (Authorization numbers 482/2016-PR and 42/2018-PR).

### Cytofluorimetric Analysis, Suppression Assay, and ELISA

In all FACS analyses, cells were treated with rat anti-CD16/32 (2.4G2) for 5 min at 4°C for the blockade of Fc receptors before assaying on a Fortessa flow cytometer and analyzed by FlowJo analysis software (Tree Star, OR, USA). Phenotypic characterization of CD4^+^ T cells and DCs derived from MLN and bone marrow, respectively, was performed by extracellular staining using the following Abs: APC-CD3 (145-2C11; Biolegend), PE-DazzleTM-B220 (RA3-6B2; Biolegend), BV510-CD4 (RM4-5; BD Horizon), PerCP-Cy5.5-CD8 (53.6.7; BD Pharmingen), BV786-CD44 (IM7; BD Horizon), APC-Cy7-CD25 (PC61; Biolegend), BV421-LAP (TW716B4; BD Horizon, CA), BV786-B220, APC-CD317 (eBio927; eBioscience), CD19 APC-Cy7 (65D; Biolegend), ef780-CD11c (N418; eBioscience), BV510-MHCII, ef450-CD11b (M1/70; eBioscience); BV711-F4/80 (T45-2342; BD Biosciences). PE-Foxp3 (FJK-16s; eBioscience) was used for the intracellular determination of Foxp3. Unconjugated polyclonal rabbit anti-mouse/human NCOA7 (Thermo Fisher Scientific) was used in combination with donkey anti-rabbit IgG DyLight™ 488 (Poly4064, Biolegend) to determine the intracellular expression of NCOA7 in MLN, HEK293, and MEF cells. Mouse AhR protein expression was evaluated in MEF cells using an AhR specific antibody (4MEJJ; eBioscience). For suppression assays, naive CD4^+^CD25^−^ cells (2 × 10^5^) were cultured for 3 days with various numbers of CD4^+^ T cells preincubated with 50 μM 3-HAA, Biot1, or Biot2 for 48 h in the presence of soluble anti-CD3 (1 μg/ml; affinity-purified clone 2C11) (39). Proliferation was measured by incorporation of carboxyfluorescein diacetate succinimidyl ester (CFSE) according to standard procedures. Mouse transforming growth factor-β (TGF-β1) was measured in culture supernatants by ELISA using a specific kit (R&D Systems), as described (40).

### Pull-Down Experiments, 2-D Electrophoresis, Matrix-Assisted Laser Desorption/Ionization Time-of-Flight Mass Spectrometry (MALTI-TOF-MS) Analysis, and Database Search

Thirty × 10^6^ MLN cells were lysed in RIPA buffer in the presence of protease inhibitors, incubated with Biot3 (as a control), or Biot2 at 300 μM, and immunoprecipitated with streptavidin-agarose (Sigma). Freshly prepared immunoprecipitate (IP) samples were stored at −80°C before being sent on dry ice to Applied Biomics for proteomics analysis. 2-D electrophoresis, matrix-assisted laser desorption/ionization time-of-flight mass spectrometry (MALTI-TOF-MS) analysis, and database search were performed by Applied Biomics as detailed in Supplementary Methods.

### Generation and Expression of *Ahr* and *Ncoa7* Gene Constructs

The construct expressing *Ahr* was purchased from Genecopeia and that expressing *Ncoa7* was generated from the cDNA of cDCs stimulated with IFN-γ. Briefly, the *Ncoa7* coding sequence was cloned in the pEM_02 vector, by adding *Spe*I and *Not*I restriction sites to the *Ncoa7* 5′ and 3′ region, respectively. For both *Ahr* and *Ncoa7* constructs, 5 μg DNA was used to transfect 1 × 10^6^ MEF or HEK293 cells by lipofectamine 3000 (Invitrogen). Cells transfected with the empty plasmid were used as a control.

### Measurements of AhR Activation

To assess the activation of AhR, we used mouse hepatoma cells (H1L1.1c2), containing the stably integrated AhR xenobiotic responsive element driven by a firefly luciferase reporter plasmid, pGudLuc6.1 (41). Cells were seeded in 96-well plates at a density of 100 × 10^5^ cells in 200 μl. After 12 h at 37°C, cells were stimulated for 6 h with increasing concentrations of Kyn, as the endogenous reference AhR ligand, in the presence or absence of different concentrations of 3-HAA before lysis. To evaluate the role of NCOA7 in 3-HAA activity, mouse embryonic fibroblasts (MEFs), constitutively expressing AhR, were transfected with a firefly luciferase reporter plasmid containing an upstream enhancer of mouse *Cyp1a1*, a gene upregulated by AhR activation, after transfection with the *Ncoa7*-encoding plasmid. Mock-transfected cells were used as a control. Luciferase assays were performed using luciferase reporter assay kit (Promega). The *Renilla* luciferase activity was measured, and results are presented as fold induction.

### Gene Silencing, Real-Time RT-PCR, and Western Blotting

Real-Time RT-PCR (for *Ncoa7* and *Actb*, coding for β-actin) analyses were carried out as described (39), using primers listed in Supplementary Table 1. Data were calculated as the ratio of gene to *Actb* expression by the relative quantification method (ΔΔCT; means ± SD of triplicate determination), and data are presented as normalized transcript expression in the samples relative to normalized transcript expression in control cultures (in which fold change = 1; dotted line). AhR protein expression was investigated by immunoblot with a goat polyclonal anti-mouse/human antibody (R&D System). NCOA7 expression was investigated with a rabbit polyclonal anti-mouse/human NCOA7 antibody (Thermo Fisher Scientific). Anti–β-tubulin was from Sigma-Aldrich. Densitometric analysis of specific signals was performed within a linear range of blot exposure, in each experiment selecting the two lowest-exposure times required for detecting signals.

### Gene Expression Data of Mouse and Human Immune Cells

Raw gene expression data of immune cells and DCs, obtained using Mouse Gene 1.0 ST arrays, were downloaded from Gene Expression Omnibus GSE15907 (https://www.ncbi.nlm.nih.gov/geo/query/acc.cgi?acc=GSE15907). Accession numbers of samples used in this study are listed in Supplementary Table 2. Probe level signals were converted to expression values using robust multi-array average procedure RMA (42) of the Bioconductor *affy* package and a custom definition file for mouse Gene 1.0 ST arrays based on Entrez genes (http://brainarray.mbni.med.umich.edu/Brainarray/Database/CustomCDF/23.0.0/entrezg.asp) (43). Raw gene expression data of immune cells isolated from healthy human blood have been downloaded from Gene Expression Omnibus GSE28490 and GSE28491. Accession numbers of samples used in this study are listed in Supplementary Table 3. Probe level signals were converted to expression values using robust multi-array average procedure RMA (42) of the Bioconductor affy package and a custom definition file for Affymetrix Human Genome U133 Plus 2.0 arrays based on Entrez genes (hgu133plus2hsentrezgcdf version 21.0.0; http://brainarray.mbni.med.umich.edu/Brainarray/Database/CustomCDF/21.0.0/entrezg.asp) (43).

All data analyses were performed in R version 3.5.1 using Bioconductor libraries and R statistical packages.

### Statistical Analyses

All analyses were performed using Prism version 6.0 (GraphPad Software). Data were analyzed by 2-tailed unpaired Student's *t* test or 2-way ANOVA followed by *post hoc* Bonferroni's test, when three or more samples were under comparison, respectively, using at least three values from three experiments per group. Differences were considered significant with *p* < 0.05. Data are representative of two-three independent experiments.

## Results

### Generation of Biologically Active, Biotin-Tagged 3-HAA

In order to identify the molecular targets of 3-HAA, we resorted to affinity-based proteomics, also known as a pull-down methodology, one of the most widely applied methodologies to identify targets of biologically active compounds. In pull-down experiments, biotin is often used for generating molecular probes (44). 3-HAA is characterized by the presence of three functional groups—carboxyl, amino, and hydroxyl—each potentially taggable by biotin. In the absence of indications of its direct target and thus of a possible structure-activity relationship, we attempted the biotinylation of 3-HAA in each of its functional moieties. 3-HAA was successfully biotinylated using a C6 spacer in either the carboxyl or hydroxyl but not amino group, leading to the generation of Biot1 and Biot2 compounds (Figure 1A and Figure S2). In order to understand whether the amino group could be nevertheless important in the biological activity of 3-HAA, an additional biotinylated compound, i.e., Biot3, was generated by tagging the hydroxyl group of 3-hydroxybenzoic acid, a compound structurally similar to 3-HAA but lacking the amino group (Figure 1A and Figure S2).

Because the introduction of a biotin moiety could have influenced the biological activity of native 3-HAA (34), all biotinylated compounds were characterized for their immunoregulatory profile as compared to native 3-HAA, in accordance to previous data (45). To this purpose, mouse MLN cells were incubated with 3-HAA, Biot1, Biot2, or Biot3, all at the final concentration of 50 μM. After 48 h, cells were assayed for (i) coexpression of CD25 and Foxp3, a transcription factor specific for Treg cells, by cytofluorimetric analysis (Figures 1B,C), (ii) suppressive potential in a suppressor assay with CD4^+^CD25^−^ effector T cells (Figure 1D), and (iii) release of the immunosuppressive cytokine TGF-β in culture supernatants (Figure 1E). Results showed that, as expected (45), unmodified 3-HAA significantly upregulated expression of Foxp3 in the CD25^+^ subset of MLN cells (Figures 1B,C). Moreover, in the same cell cultures, the native Trp metabolite also induced a suppressive phenotype in CD4^+^ naïve T cells (Figure 1D) and the production of TGF-β (Figure 1E). Although no effect could be detected for Biot3, incubation of MLN cells with Biot1 and, more so, Biot2 determined a functional profile very similar to that induced by 3-HAA (Figures 1B–E).

Thus, our data showed that biotinylation of 3-HAA at the hydroxyl and carboxyl residue does not significantly alter the immunoregulatory properties of the Trp metabolite. Moreover, because Biot3 did not show any significant effect, our data indicated that the binding of 3-HAA to its putative target would require the presence of the amino group in its unmodified form.

### NCOA7 Is a Molecular Target of Biotin-Tagged 3-HAA

**Figure 2:**
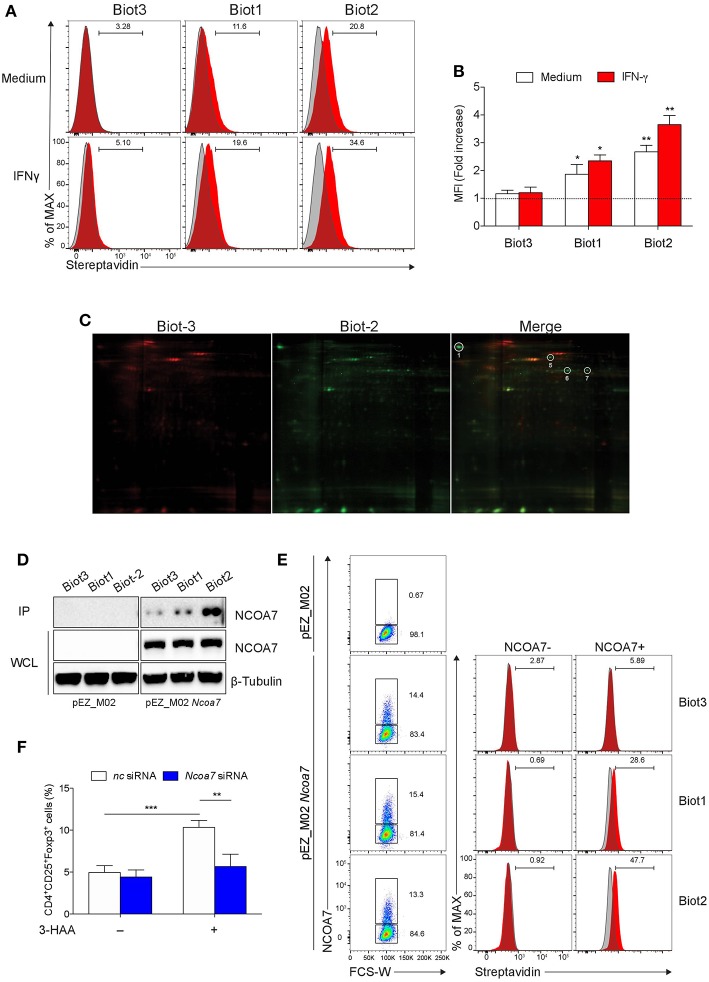
Biotinylated 3-HAA Immunoprecipitates NCOA7 in MLN cells. **(A)** Cytofluorimetric analyses of MLN cells. Cells were either incubated with medium alone or 100 U/ml IFN-γ for 24 h and then stained with 300 μM Biot1, Biot2, or Biot3 followed by Qdot 605-conjugated streptavidin (red histograms). Cell incubation with Qdot 605-conjugated streptavidin alone was used as control (gray histograms). **(B)** Mean fluorescence units (MFI) for at least three experiments conducted with the same samples as in **(A)** are presented relative to results obtained in cells stained with Qdot 605-conjugated streptavidin (dotted line, 1 fold). **(C)** Two-dimensional gel electrophoresis of protein immunoprecipitates obtained by incubation of MLN cell lysates with Biot2 or Biot3 and streptavidine-conjugated agarose. Spots selectively immunoprecipitated by Biot2 as compared to Biot3 are numbered. High confidence spots No. 1, 6, and 7, eluted from gel and subjected to MALDI-TOF-MS, are boxed. **(D)** Immunoblot analysis with anti-NCOA7 antibodies of immunoprecipitates (IP) obtained with 3-HAA biotinylated derivatives from mock-transfected (pEZ_M02) or *Ncoa7*-transfected (pEZ_M02 *Ncoa7*) HEK293 cells and of whole cell lysates (WCL) as loading control. One experiment representative of three. **(E)** Cytofluorimetric analyses of HEK293 cells transfected with the *Ncoa7*-expressing plasmid. Cells were stained with 300 μM Biot1, Biot2, or Biot3 followed by Qdot 605-conjugated streptavidin. Cell incubation with Odot 605-conjugated streptavidin alone was used as control (gray histogram). Binding of biotinylated derivatives was evaluated in NCOA7^+^ and NCOA7^−^ gated cells. **(F)** Percentages of cells coexpressing CD25 and Foxp3 cells evaluated by cytofluorometry in whole MLN cells treated either with an *Ncoa7*-specific siRNA or control siRNA, after activation with anti-CD3 and -CD28 antibodies, in the presence or absence of 50 μM of 3-HAA, Biot1, Biot2, Biot3, or medium alone (control) for 72 h. A mouse IgG2a antibody was used as isotype control. In **(B,F)**, means ± SD of three different experiments are shown. ^*^p < 0.05; ^**^p < 0.01; ^***^p > 0.001 (ANOVA followed by *post-hoc* Bonferroni's test).

Pull-down and subsequent mass spectrometric analyses of the isolated proteins have been described as the most powerful and frequently applied approach in biorecognition studies (44). Before attempting the isolation and identification of the putative 3-HAA target/s, we first investigated what conditions would be best used in pull-down experiments. To this purpose, MLN cells were examined for binding of biotinylated derivatives by cytofluorimetric analysis using PE-conjugated streptavidin. Cells were assayed either in basal conditions or after stimulation with 100 U/ml IFN-γ for 24 h. Biot3 was used as a negative control. Results showed that Biot1 and more so Biot2, but not Biot3, significantly bound MLN cells (Figures 2A,B). The highest binding (>30%) was observed in cells obtained from MLNs stimulated with IFN-γ and incubated with Biot2. The fact that Biot3 did not display any significant binding in any types of cell would exclude non-specific effects possibly occurring via the biotin and/or spacer moiety in our setting.

Based on these results, we conducted pull-down assays using Biot2 to identify 3-HAA targets in MLN cells stimulated with IFN-γ. Biot3 was used as a negative control. Cell lysates were incubated with the biotinylated 3-HAA derivatives, then with streptavidin-agarose, and the resulting immunoprecipitates were subjected to 2-D gel electrophoresis. Results showed that Biot2 specifically immunoprecipitated four proteins (spots No. 1, 5-7) in addition to those detected in Biot3 immunoprecipitates (Figure 2C and Figure S3). Among these, three proteins gave high confidence spots (No. 1, 6, and 7), i.e., sufficiently distant from others (Figure S4), and were thus subjected to gel excision and MALDI-TOF-MS analysis. As shown in Table 1, the three spots matched with high confidence to *Mus musculus* NCOA7 [previously known as ERAP140, a conserved tissue-specific nuclear receptor coactivator (46)], propionyl-CoA carboxylase β polypeptide (PCCB), and methylcrotonoyl-CoA carboxylase β chain (MCC2), respectively. At variance with NCOA7, both propionyl-CoA carboxylase and methylcrotonoyl-CoA carboxylase have often been found to occur non-specifically in protein immunoprecipitates obtained via the use of biotinylated compounds (47), an effect possibly due to their requirement for biotin as a cofactor during enzyme catalysis (48). We thus excluded propionyl-CoA carboxylase and methylcrotonoyl-CoA carboxylase from our following analyses.

**Table 1:** MALDI-TOF-MS results.

**Spot number**	**1**	**6**	**7**
**Match quality**	**High confidence**	**High confidence**	**High confidence**
Top ranked protein name (species)	Nuclear receptor coactivator 7 (*Mus musculus*)	Propionyl-CoA carboxylase β polypeptide (*Mus musculus*)	Methylcrotonyl-CoA β chain (*Mus musculus*)
Protein molecular weight (Da)	106.291	58.340	30.158
Protein pI	5.3	7.6	8.7

To further confirm the capacity of biotin-tagged 3-HAA to bind NCOA7, HEK293 cells (not expressing NCOA7) were transfected with an *Ncoa7* expressing vector, lysates were incubated with the biotinylated derivatives, and the obtained immunoprecipitates were immunoblotted with an anti-NCOA7 specific antibody. Mock-transfected HEK293 cells (i.e., transfected with the empty vector) were used as a control. Results showed that NCOA7 expression can be detected in whole cell lysates of *Ncoa7*- but not mock-transfected cells (Figure 2D). Furthermore, the antibody did recognize a 106.2 kDa protein, corresponding to NCOA7, in Biot2- and, to a lesser extent, Biot1-immunoprecipitates in *Ncoa7*- but not mock-transfected cells. The NCOA7 protein could also be revealed in Biot3 immunoprecipitates (though at a very low level), suggesting that the lack of the amino residue in 3-HAA does not totally impair the binding of the Trp metabolite to the nuclear activator, at least in cells overexpressing NCOA7. Binding of Biot2 to NCOA7 was also assessed in *Ncoa7*-transfected HEK293 cells by cytofluorometric analysis using an NCOA7-specific antibody. Results showed a significant binding of Biot1 and Biot2, but not Biot3, in NCOA7^+^ but not NCOA7^−^ cells (Figure 2E).

To evaluate the functional role of NCOA7 in mediating immunoregulatory effects by 3-HAA, MLN cells, previously treated with *Ncoa7* specific or control siRNA, were incubated with native 3-HAA as in Figure 1B and then tested for the generation of Foxp3^+^ cells and production of TGF-β. Silencing of the NCOA7-expressing gene (Figure S5A) determined a significant reduction in the induction of CD25^+^Foxp3^+^ cells (Figure 2F and Figure S5B) and release of TGF-β (Figure S5C).

Therefore, these data further confirmed the protein binding ability of biotin-tagged 3-HAA and, most importantly, identified a coactivator of nuclear receptors as functional mediator of an immunoregulatory Trp metabolite.

### NCOA7 Binding by 3-HAA Increases AhR Transcriptional Activity

Nuclear receptors are a class of ligand-activated transcriptions factors regulated by coactivators and co-repressors, i.e., molecules capable of increasing or reducing the transcription factor activity, respectively (49, 50). In general, coactivators serve as a bridge between DNA-bound transcription factors and RNA polymerase II, exploiting mechanisms such as recruitment of histone acetyl transferases and consequent opening of the chromatin structure (51). Though demonstrated to be an authentic coactivator, NCOA7 has little structural homology with other nuclear receptor cofactors (46) and its specific physiological function has not been completely understood.

Interestingly, interactome databases (i.e., http://www.ebi.ac.uk/intact/) indicate that NCOA7 may interact with a discrete number of proteins, including the ligand-activated transcription factor AhR. As a matter of fact, NCOA7 was previously immunoprecipitated with AhR in human breast cancer cells using AhR-specific antibodies. Moreover, in the same study, the transactivating effect of TCDD on AhR was found to increase in cells overexpressing NCOA7 (52). In another study, NCOA7 promoted tumor cell proliferation via enhancement of AhR transcriptional activity in the absence of externally added ligands, possibly suggesting that NCOA7 is a ligand-independent coactivator of AhR (53). However, these data did not exclude the possible role played by endogenous molecules.

**Figure 3:**
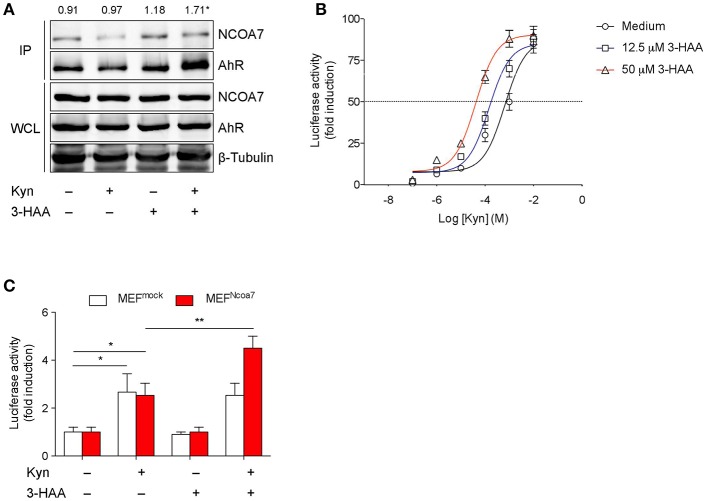
3-HAA Increases AhR Transcriptional Activity induced by Kyn in NCOA7^+^ Cells. **(A)** HEK-293 cells cotransfected with plasmids encoding for NCOA7 and AhR were incubated with 50 μM Kyn and 3-HAA, either alone or in combination. After 4 h, protein extracts were used for immunoprecipitation with the anti-NCOA7 antibody. Immunoprecipitated (IP) proteins were analyzed by Western blotting with anti-AhR antibodies. Whole cell lysates (WCL; 10% of the input) were used as loading control. Upper values indicate the means of AhR (IP):AhR (WCL) ratios that were calculated by densitometric quantification of the specific bands detected in three independent experiments.^*^p < 0.05 (kynurenine and 3-HAA co-treatment vs. unstimulated sample; two-tailed Student's *t*-test). **(B)** Transactivation activity of AhR by Kyn in the presence or absence of 3-HAA at different concentrations in H1L1 cells. **(C)** Transactivation activity of AhR by Kyn (50 μM) in the presence or absence of 3-HAA (50 μM) in MEFs, either untransfected (MEF^mock^) or transfected with the *Ncoa7* gene (MEF^NCOA7^). One experiment of three is shown. ^*^p < 0.05; ^**^p < 0.01 (ANOVA followed by *post-hoc* Bonferroni's test).

Because AhR can also be activated by endogenous ligands (24), including Kyn, and because our current data indicated the occurrence of NCOA7 binding by a Trp metabolite, we investigated whether 3-HAA could favor recruitment of NCOA7 by AhR and modulate the transactivating ability of the receptor. To this purpose, mouse AhR and NCOA7 were coexpressed in human HEK293 cells and their association was examined by reciprocal immunoprecipitation and Western blotting. Before lysis, cells were incubated with 50 μM Kyn and 50 μM 3-HAA, either alone or in combination, for 4 h. Incubation with medium in the absence of Trp metabolites was used as a negative control of immunoprecipitation, whereas immunoblotting of whole cell lysates was performed as a control of AhR and NCOA7 expressions in each sample. Results showed that, despite a comparable content of AhR and NCOA7 along all samples, cell incubation with 3-HAA but not Kyn alone induced a significant increase in AhR-NCOA7 association, an effect that was even greater in cells incubated with both Trp metabolites (Figure 3A).

We next evaluated AhR transcriptional activity in cells stimulated with Kyn in the presence or absence of 3-HAA. To this purpose, we used H1L1 cells, constitutively expressing both AhR and NCOA7 and stably transfected with a firefly luciferase reporter plasmid containing an upstream enhancer of mouse *Cyp1a1*, a gene upregulated by AhR activation (54). The enhancing effect of the Trp metabolite was tested at fixed concentrations (12.5 and 50 μM) over a concentration/effect curve of Kyn in activating AhR. Results showed that the co-presence of 3-HAA induced a shift of to curve to the left, in a dose-dependent fashion, thus suggesting an increase of Kyn affinity in AhR binding (Figure 3B). These data also indicated that the enhancing effects of 3-HAA are also detectable at lower concentrations of the metabolite, i.e., 12.5 μM, more compatible with physiological levels. Notably, no AhR transcriptional activation could be observed with 3-HAA, i.e., in the absence of Kyn, at both concentrations (Figure 3B and Figure S6A).

To evaluate the functional role of NCOA7 in 3-HAA–driven enhancement of AhR activation, we conducted experiments with MEFs, which basally express AhR but not NCOA7, after transfection with the *Ncoa7*-expressing vector or vector alone (mock) (Figures S6B,C). Resulting MEF^mock^ and MEF^NCOA7^ cells were incubated with medium alone, 50 μM Kyn, 50 μM 3-HAA, or a combination of the two compounds. After 6 h, *Cyp1a1* transcriptional activity was increased in either type of cell incubated with Kyn but not 3-HAA alone (Figure 3C). The absence of agonist activity in cells incubated with 3-HAA alone did therefore exclude the possibility that, at least in our experimental conditions, 3-HAA had been converted into cinnabarinic acid, a Trp metabolite capable of direct AhR agonist activity (23). Moreover, the absence of difference in the AhR activation by Kyn in MEF^NCOA7^ vs. MEF^mock^ cells would exclude the possible role of endogenous production of 3-HAA. Importantly, the co-presence of 3-HAA and Kyn led to a greater fold-increase of *Cyp1a1* transcriptional activity in MEF^NCOA7^ as compared to MEF^mock^ cells.

As a whole, our data indicated that the Trp metabolite 3-HAA, despite being incapable of activating AhR on its own, binds NCOA7 and, in consequence of that, promotes a physical interaction of the nuclear coactivator with AhR and enhanced AhR transcriptional activation by Kyn.

### Conventional DCs Express High Levels of NCOA7

At variance with AhR, characterized by a wide distribution in the body, NCOA7 expression is both tissue- and cell-type specific and has been shown to be mostly abundant in brain neurons and some tumors (46). A more recent semiquantitative analysis unveiled that the *Ncoa7* gene is also expressed in mouse spleen (55), although the cells specifically expressing NCOA7 were not identified. An analysis of conserved genetic signatures in lymph node resident DCs indicated that the gene coding for NCOA7 appears to be selectively expressed (though at a low level) in both mouse and human DCs (56).

**Figure 4:**
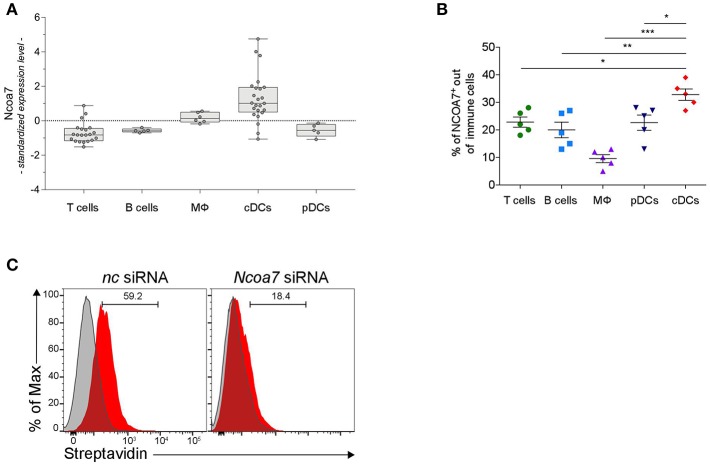
DCs Express High Levels of NCOA7. **(A)** Expression of *Ncoa7* transcript in different types of immune cells from mouse lymph nodes. *p* < 0.0001 (one way ANOVA). **(B)** Percentages of NCOA7^+^ cells measured by cytofluorometric analysis of NCOA7 expression in gated (CD3^−^B220^+^CD19^+^ for B cells; B220^−^CD11c^−^MHCII^−^CD3^+^ for T cells; B220^−^BST2^−^CD11c^−^CD11b^+^F4/80^+^ for macrophages, MΦ; CD3^−^ CD19^+^B220^+^BST2^+^ for pDCs; and CD3^−^B220^−^ CD19^−^BST2^−^MHCII^+^ CD11b^+^CD11c^+^ for cDCs) immune cells from MLNs. Rabbit IgG was used as isotype control. ^*^p < 0.05; ^**^p < 0.01; ^***^p < 0.001 (unpaired Student's *t*-test). **(C)** Cytofluorimetric analyses of cDCs gated from MLNs as in **(B)** after treatment with *Ncoa7*-specific or negative control (nc) siRNA and incubation with Biot2 followed by Qdot 605-conjugated streptavidin.

In order to have a more comprehensive evaluation of NCOA7 expression in organs and cells of the immune system, we conducted a meta-analysis of public microarray data restricted to the expression of the *Ncoa7* gene in mouse lymph nodes and spleens in distinct cell types, i.e., CD4^+^ and CD8^+^ T lymphocytes, B cells, macrophages, natural killer (NK) cells, CD11c^+^ conventional DCs (cDCs), and plasmacytoid DCs (pDCs). Results showed that *Ncoa7* is highly expressed in cDCs but not pDCs or other cell types (Figure 4A and Figure S7A). Similarly, the highest expression of *NCOA7* transcripts could be observed in human cDCs, although in this species the plasmacytoid DC subset also express high levels of the nuclear coactivator (Figure S7B). Cytofluorimetric analyses confirmed the high percentage of NCOA7^+^ cells in gated cDCs as compared to other gated cells of mouse MLNs (Figure 4B). In order to evaluate whether 3-HAA could bind NCOA7 in cells physiologically expressing high levels of the nuclear coactivator, MLN cells were treated with *Ncoa7*-specific or control siRNA prior incubation with Biot2 and cytofluorometric analysis of gated cDCs. Results showed that the silencing of the *Ncoa7* gene abrogated Biot2 binding in cDCs from MLNs (Figure 4C).

As a whole, our data indicated that cDCs represent the immune cells that express the highest levels of NCOA7 and that bind 3-HAA in an NCOA7-dependent fashion.

### 3-HAA Potentiates the Induction of Immunoregulatory DCs in an NCOA7-Dependent Fashion

**Figure 5:**
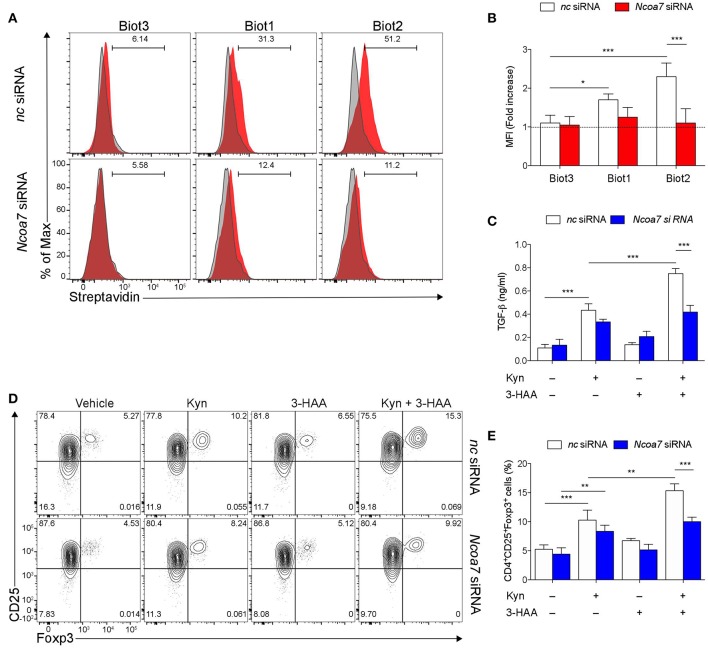
NCOA7-expressing DCs increase their immunoregulatory potential in the co-presence of Kyn and 3-HAA. **(A)** Purified BM cDCs, pretreated with either *Ncoa7*-specific or negative control (nc) siRNA, were incubated with 300 μM Biot1, Biot2, or Biot3 followed by Qdot 605-conjugated streptavidin. Cell incubation with Qdot 605-conjugated streptavidin alone was used as a control (gray histogram). One experiment representative of three, whose means are shown in **(B)**. **(C)** Production of TGF-β1 by cDCs, transfected with either control or *Ncoa7*-specific siRNA, after incubation with Kyn and/or 3-HAA, both at 50 μM, for 48 h. **(D)** Representative cytofluorimetric dot plots of CD25 and Foxp3 coexpression in CD4^+^ cells co-cultured for 48 h with cDCs (either transfected with control or *Ncoa7*-specific siRNA) preincubated for 24 h with 3-HAA or Kyn, both at 50 μM. A mouse IgG2a antibody was used as isotype control. Shown in upper right quadrants are percentages of double-positive cells. One experiment representative of three, whose means are shown in **(E)**. ^*^p < 0.05; ^**^p < 0.01; ^***^p > 0.001 (ANOVA followed by *post-hoc* Bonferroni's test).

Trp metabolites are known to play a critical role in conferring immunoregulatory functions on DCs, including generation of Treg cells and production of TGF-β (32, 57). In order to carry out functional experiments with adequate numbers of purified cDCs, we generated *in vitro* cDCs from the BM. Then, sorted cDCs (Figure S8A) were analyzed for NCOA7 expression. Similarly to what was found in LNs and spleens, we confirmed that also cDCs differentiated from BM cultures expressed NCOA7 (Figure S8B). Cytofluorimetric analyses confirmed a high percentage of binding of Biot2 and Biot1 but not Biot3 in BM cDCs preincubated with negative control but not *Ncoa7*-specific siRNA (Figures 5A,B). To evaluate the effects of NCOA7 engagement by 3-HAA in conferring immunoregulatory properties on DCs, BM cDCs, either transfected with an *Ncoa7*-specific or a control siRNA, were incubated with Kyn and/or 3-HAA. cDCs were either cultured alone, to determine their release of TGF-β1 in culture supernatants, or with naïve CD4^+^CD25^−^ T cells, to evaluate the DC capacity to induce Foxp3 expression in T cells. Results showed that Kyn but not 3-HAA alone significantly increased the TGF-β1 production by cDCs (Figure 5C) and Foxp3 expression in CD25^+^ T cells (Figures 5D,E), effects that were not dependent by NCOA7 expression. The DC pretreatment with both Kyn and 3-HAA led to a further significant increase in both TGF-β1 production by cDCs (Figure 5C) and Foxp3 expression in CD25^+^ T cells (Figures 5D,E), but only if NCOA7 was expressed by the cDCs.

Thus, these data indicated that the co-presence of Kyn and its metabolite 3-HAA in cell microenvironments enhances the immunoregulatory functions of NCOA7^+^ cDCs.

## Discussion

Nuclear receptors are a class of ligand-activated transcription factors that appeared early during metazoan evolution (58). All transcription factors, irrespective of structure or function, recruit protein complexes consisting of regulators of gene transcription, i.e., coactivator and corepressor proteins. The intrinsic functions of transcription factors are therefore modulated by the recruitment of these ancillary factors, which favor the interaction with histone acetyl transferase (HAT; for coactivators) or histone deacetylase (HDAC; corepressors) enzymes that promote or inhibit gene transcription, respectively (59, 60).

Although not belonging to the class of nuclear receptors, AhR is an ancient transcription factor whose evolution has pushed toward the need of an agonist for its activation and of complex interactions with other molecular partners in high vertebrates (61, 62). After ligand binding, AhR translocates to the nucleus, where it dimerizes with the AhR nuclear translocator (ARNT) protein. The AhR/ARNT dimer binds to xenobiotic responsive elements (XREs) located in the 5′ flanking region of the *Cyp1a1* gene, leading to the activation of its transcription. Although no AhR corepressor has been identified to date, some evidence has been provided for the presence of coactivators in the enhancesome of AhR—namely, steroid receptor coactivator 1 (SRC-1), NCOA2 (also belonging to the SRC family), and p/CIP—all of which increase TCDD-dependent expression of a XRE-driven reporter gene in hepatoma cells (62). However, the identity of other factors and the mechanisms by which they are recruited by the AhR transcriptional complex to its cognate response element are largely unknown (63). Moreover, no information is available yet for the role of any coactivator in enhancing AhR effects in immune regulation.

In the present work, by using biorecognition studies, we identified NCOA7 as a molecule bound by 3-HAA, a Trp metabolite produced downstream IDO1 along the kynurenine pathway. Engagement of NCOA7 by 3-HAA favored the interaction of this nuclear coactivator with AhR and increased AhR activation in transfected cells. Physiologically, the conditioning of NCOA7^+^ but not NCOA7^−^ cDCs with a combination of an endogenous AhR agonist, i.e., Kyn, and 3-HAA enhanced the capacity of cDCs to produce immunosuppressive TGF-β and increase the induction of CD4^+^CD25^+^Foxp3^+^ Treg cells. Although not clarified in terms of molecular mechanism/s, the potentiating effects of 3-HAA in the immunoregulatory profile of IDO1^+^ DCs have already been described (20). More specifically, Xie et al. found that IDO1^+^ DCs effectively suppress the proliferation of CD4^+^CD25^−^ T cells *in vitro* and that this effect could be enhanced by adding 3-HAA. Moreover, in an allogeneic small bowel transplantation model, ratios of Kyn/Trp in the blood (i.e., an indicator of systemic Trp metabolism) of 3-HAA– treated mice were found to increase without IDO1^+^ DC transfusion. Because AhR activation can upregulate IDO1 expression in DCs (29, 32), the *in vivo* data obtained by Xie et al. could be explained on the basis of an NCOA7-mediated, AhR enhancing mechanism induced by 3-HAA.

Although NCOA7 can be recruited to the promoter region of ERα target genes following estradiol treatment, NCOA7 has a lesser extent of sequence and structure homology with other coactivators, including those belonging to the SRC family, and might function as a distinct coactivator class (46). Several gene polymorphisms of NCOA7 have been associated with the development of breast cancer (64, 65). In another study, NCOA7 was identified as a potential biomarker in oral squamous cell carcinoma (53). Interferon β1b, commonly used as therapeutic agent in multiple sclerosis (i.e., an inflammatory/autoimmune disease) induces the expression of an alternative-start transcript of the human *NCOA7* gene, which may be involved in the protection from inflammatory oxidative stress (66). More recently, the same interferon-inducible isoform of NCOA7 was found to mediate immunoregulatory effects in antigen presenting cells in a model of viral infection (67). Therefore, although its functions have not been completely understood, NCOA7 may contribute to immunoregulatory mechanisms that can be either pathogenetic (i.e., in neoplasia) or protective (i.e., in autoimmunity), similarly to AhR (68, 69).

Activation of nuclear receptors by specific agonists leads to the exposure of conserved helical sequences that allow the interaction with nuclear coactivators and thus the enhancement of gene transcriptional expression (70). No ligands directly binding coactivators or corepressors and capable of promoting their interaction with nuclear receptors have been reported so far. Nevertheless, in an important study aimed at evaluating the structure of the complex formed by human NCOR2 (i.e., a corepressor) and HDAC3, the authors identified a third party, i.e., an inositol tetraphosphate molecule, which acts as an “intermolecular glue” between the two protein partners and contributes to the activation of HDAC3 (71). Therefore, 3-HAA, besides increasing the interaction between NCOA7 and AhR by a still undefined mechanism, may also act as an “intermolecular glue” between NCOA7 and HAT enzymes, thus favoring AhR transcriptional activation of gene expression.

AhR and its nuclear translocator ARNT are expressed in most if not all cells and tissues of the body, including immune and tumor cells (26). In contrast, unlike most reported coactivators, NCOA7 exhibits both cell- and tissue-specific expression (46). NCOA7 is in fact abundant in the brain, with its expression restricted to neurons of specific cerebral areas, and is also expressed in cortex cells of kidney and in several but not all tumors. In contrast, NCOA7 is undetectable in intestine, muscle, and liver, in which AhR plays a major role in the detoxification of xenobiotics (46). Our data further emphasized these observations indicating that, among immune cells, cDCs are unique by virtue of their very high expression of NCOA7. Therefore, the restricted NCOA7 expression combined with the exposure to Trp metabolites may selectively activate AhR in cDCs, thus potentiating their immunoregulatory effects. Because NCOA7 is not expressed at the same level by all DC subsets—characterized by specific markers but also by functional profiles—our data would indicate that NCOA7 may represent a new marker of specific DC subsets, particularly prone to acquire a strong immunoregulatory profile in microenvironments enriched with Trp metabolites.

## Data Availability

All datasets generated for this study are included in the manuscript/Supplementary Files.

## Author Contributions

FF and UG designed and supervised the study as a whole. VC and OT designed and supervised the chemical part. MG performed the majority of experiments. CVa performed immunoprecipitation experiments that led to the NCOA7 identification. SM synthesized the biotinylated compounds. GS and GMa performed luciferase assays. GMo, MP, CO, CVo, RB, DM, AI, EPa, EPr, and II helped with some experiments and/or provided reagents. EM and SB performed meta-analyses of gene expression data. MB helped with the generation of gene constructs. PP and UG wrote the manuscript.

### Conflict of Interest Statement

The authors declare that the research was conducted in the absence of any commercial or financial relationships that could be construed as a potential conflict of interest.
