# Supplementary material for: Engagement of Nuclear Coactivator 7 by 3-Hydroxyanthranilic Acid Enhances Activation of Aryl Hydrocarbon Receptor in Immunoregulatory Dendritic Cells
Source: Front Immunol. 2019 Aug 20;10:1973. doi: 10.3389/fimmu.2019.01973 (PMC6710348; doi:10.3389/fimmu.2019.01973)
Supplement: Supplementary file 1 [file Data_Sheet_1.PDF]

SUPPLEMENTARY INFORMATION

Engagement of Nuclear Coactivator 7 by 3-Hydroxyanthranilic Acid Enhances Activation of Aryl Hydrocarbon Receptor in Immunoregulatory Dendritic Cells

Marco Gargaro, Carmine Vacca, Serena Massari, Giulia Scalisi, Giorgia Manni, Giada Mondanelli, Emilia M.C. Mazza, Silvio Bicciato, Maria T. Pallotta, Ciriana Orabona, Maria L. Belladonna, Claudia Volpi, Roberta Bianchi, Davide Matino, Alberta Iacono, Eleonora Panfili, Elisa Proietti, Ioana Maria Iamandii, Violetta Cecchetti, Paolo Puccetti, Oriana Tabarrini, Francesca Fallarino,* & Ursula Grohmann*

***Ursula Grohmann**, Ph.D. (ursula.grohmann@unipg.it)

***Francesca Fallarino**, Ph.D. (francesca.fallarino@unipg.it)

University of Perugia
Department of Experimental Medicine
Piazzale Gambuli, n. 1 (C Bldg, 4th Fl)
Perugia 06132, Italy

This Supplementary File includes:

- Supplementary Figures 1 to 8 with Legends
- Supplementary Tables 1 to 3
- Supplementary Methods

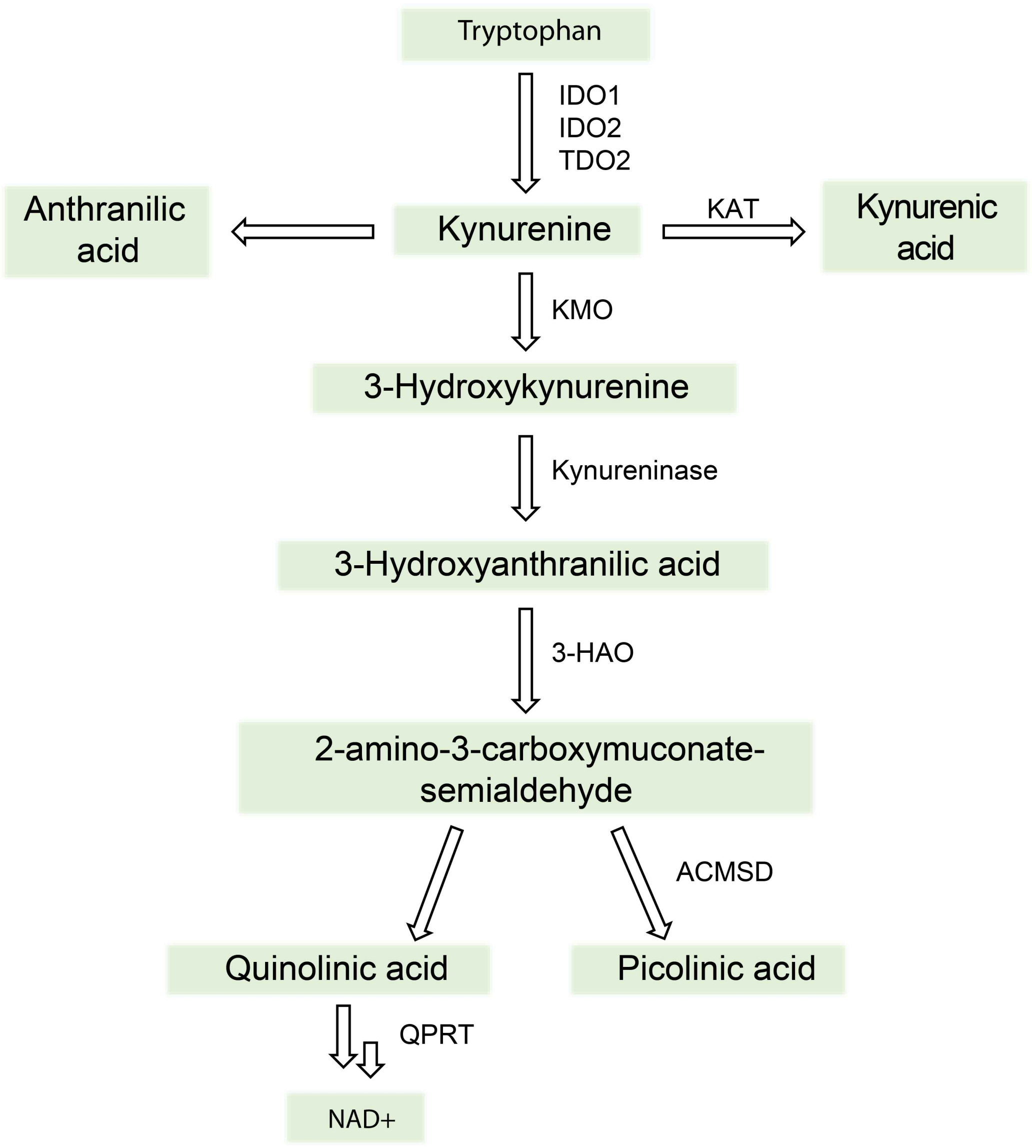

Figure S1 | Trp metabolism along the kynurenine pathway. Abbreviations: ACMSD, aminocarboxymuconate semialdehyde decarboxylase; 3-HAO, 3-hydroxyamino oxidase; IDO1 and IDO2, indoleamine 2,3-dioxygenases 1 and 2; KAT, kynurenine aminotransferase; KMO, kynurenine 3-monooxygenase; QPRT, quinolinate phosphoribosyltransferase; TDO, tryptophan 2,3-dioxygenase.

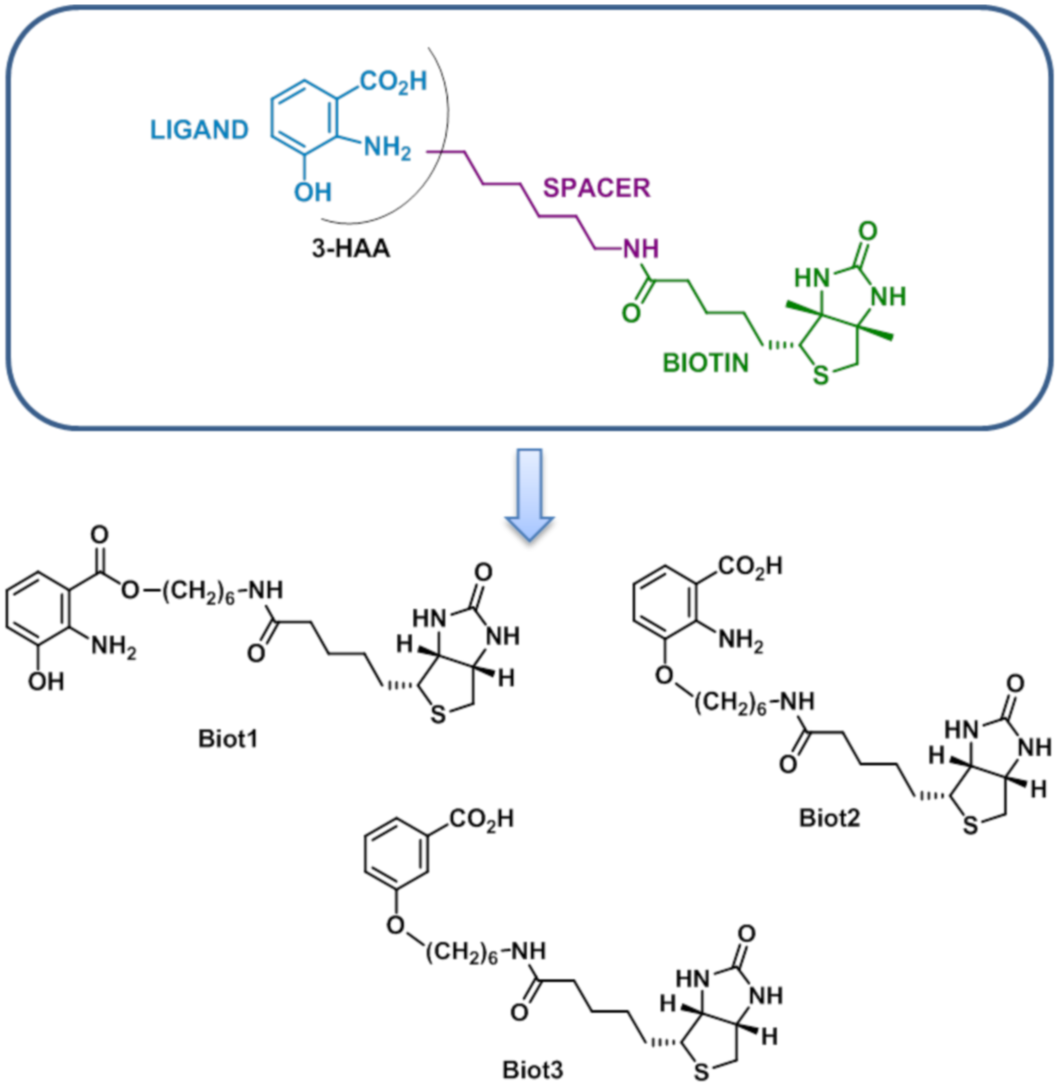

Figure S2 | Full structure of biotinylated derivatives of 3-HAA. A hexamethylenic unit (i.e., C6) was inserted as a spacer arm between the ligand and the biotin molecule to minimize steric hindrance.

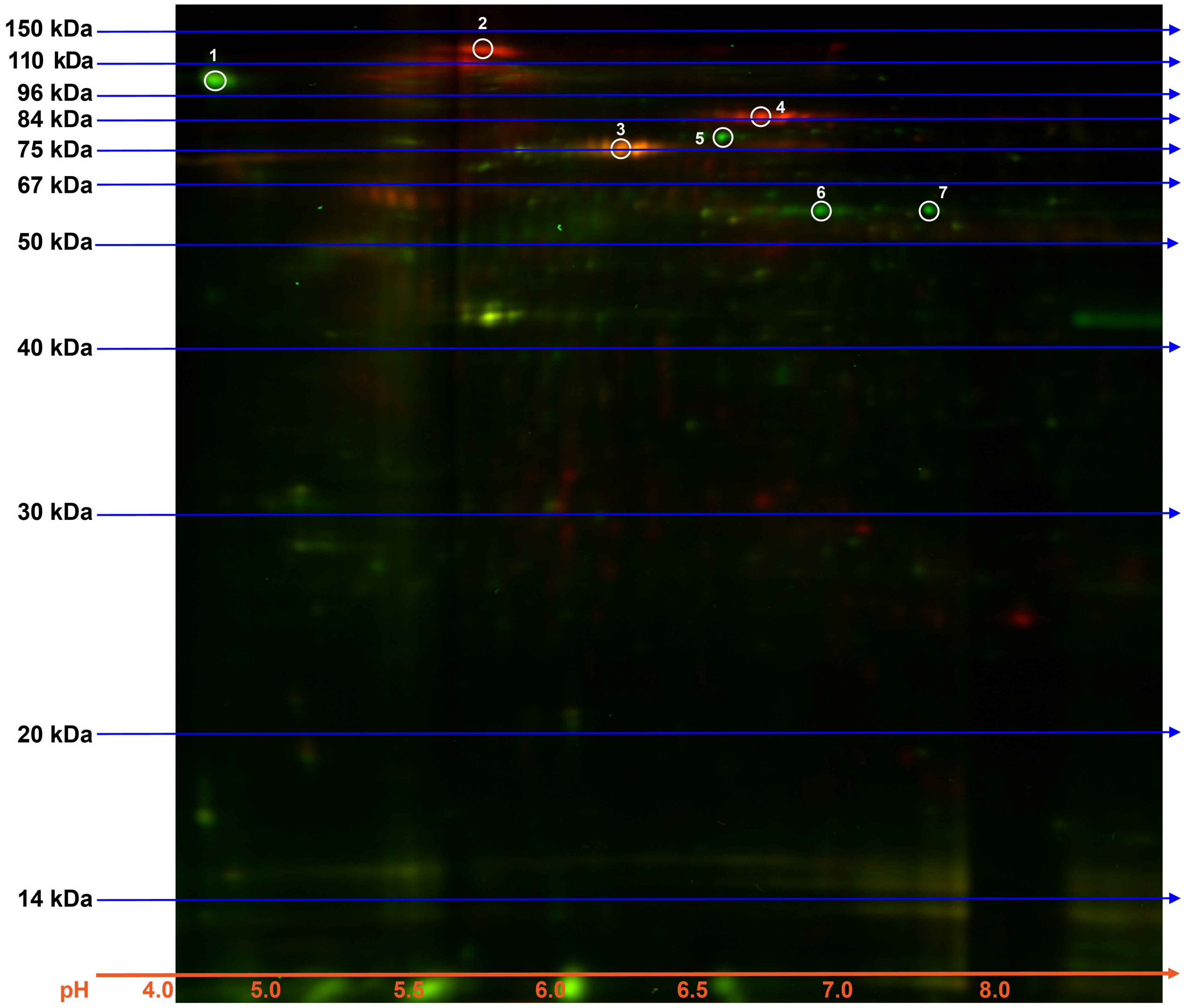

Figure S3 | Enlargement of merge of two-dimensional gel electrophoresis of protein immunoprecipitates shown in Fig. 2C. Spots selectively immunoprecipitated by Biot2 are shown in green.

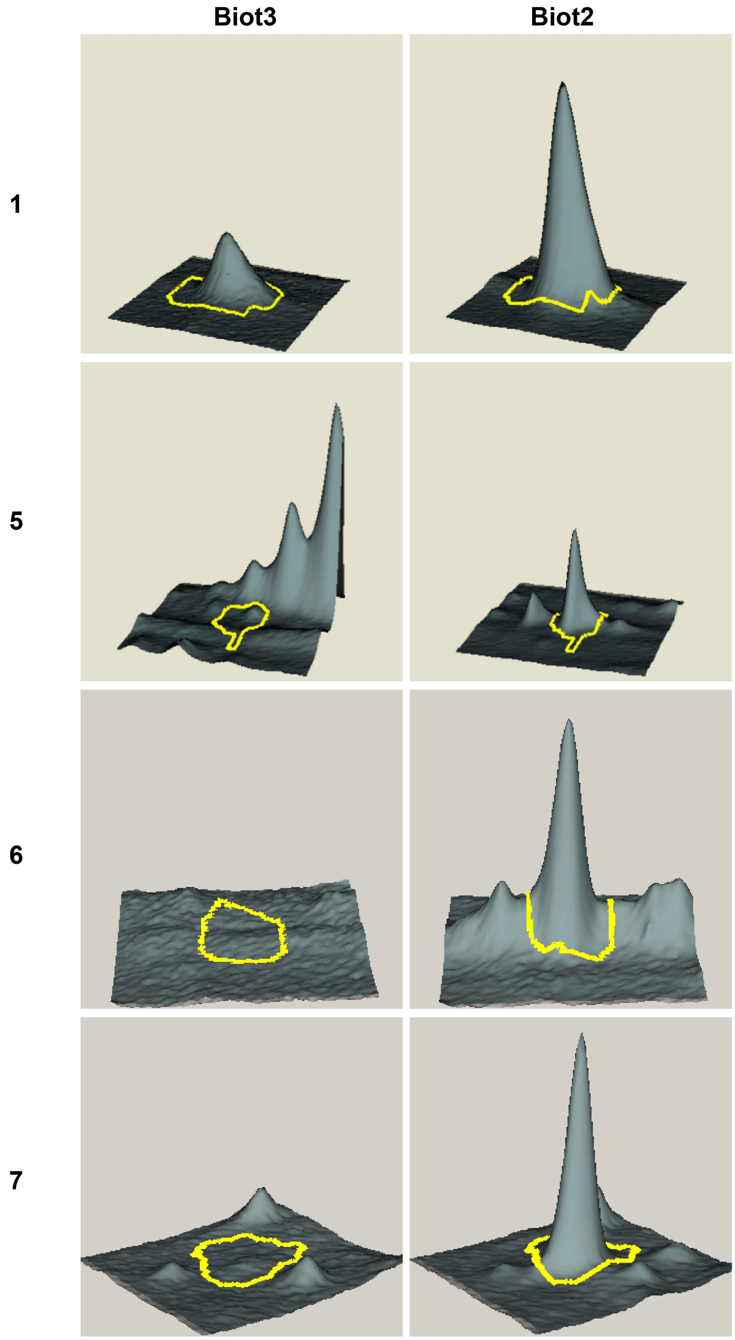

Figure S4 | 3D View of Spots No. 1 and 5-7 of Figure 2C and S3.

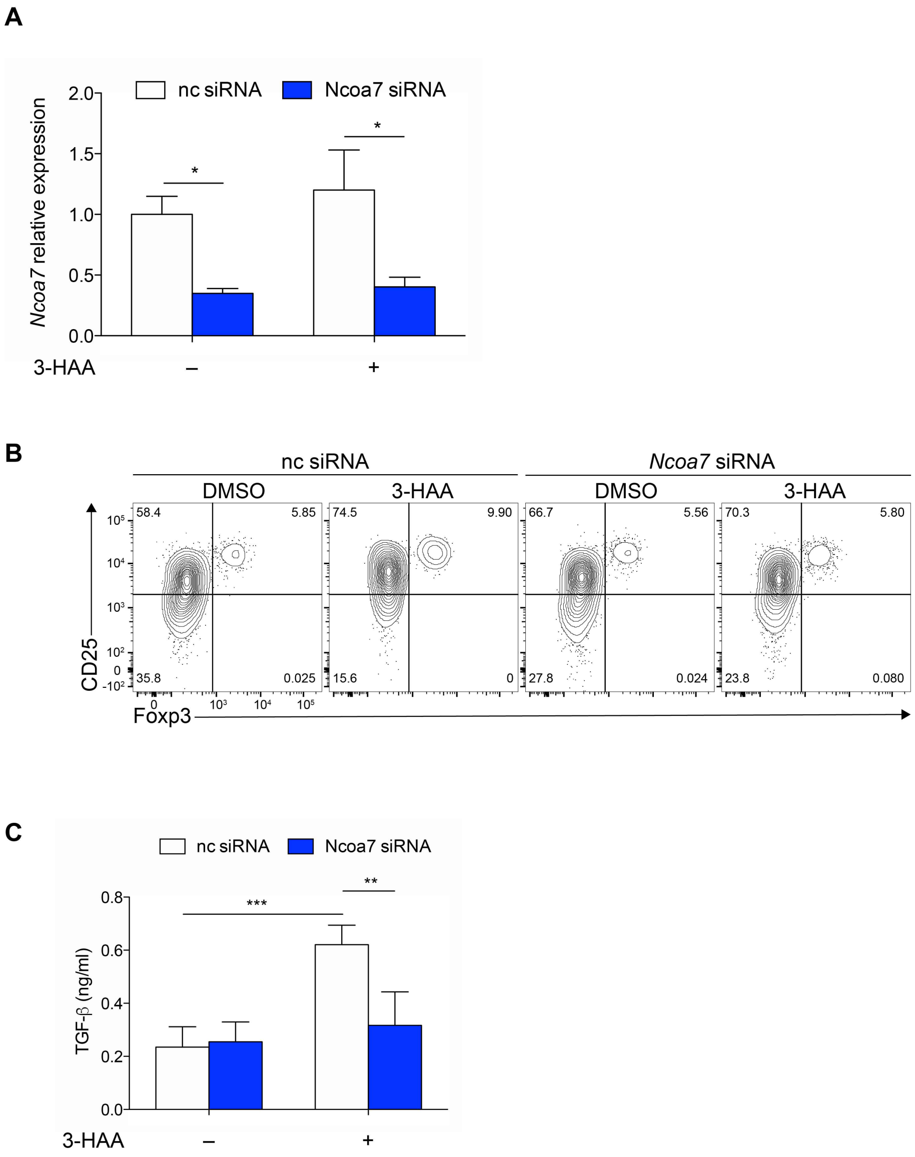

Figure S5 | NCOA7 requirement for the immunoregulatory effect of 3-HAA in MLN cells. (**A**) Efficiency of *Ncoa7* silencing in MLN cells. RT-PCR expression of *Ncoa7* transcripts was evaluated in MLN cells after transfection with a control or *Ncoa7*-specific siRNA followed by treatment with 3-HAA. Data presented are relative to transcript expression reported as means ± SD ($n = 2$; *$p < 0.05$, two-tailed Student's t-test). (**B**) Representative dot plots of CD25 and Foxp3 co-expression in $CD4^+$ cells, evaluated by FACS in whole MLN cells treated with a control or *Ncoa7*-specific siRNA. After activation with anti-CD3 and -CD28 antibodies followed by incubation 50 µM 3-HAA or medium alone (control), Foxp3 expression was assessed by intracellular staining. A mouse IgG2a antibody was used as isotype control. Shown in upper right quadrant are percentages of double-positive cells. One experiment representative of three. (**C**) TGF-β levels in supernatants of cultures established as in (**B**). Means ± SD of three different experiments are shown. *$p < 0.05$; **$p < 0.01$; ***$p < 0.001$ (ANOVA followed by post-hoc Bonferroni's test).

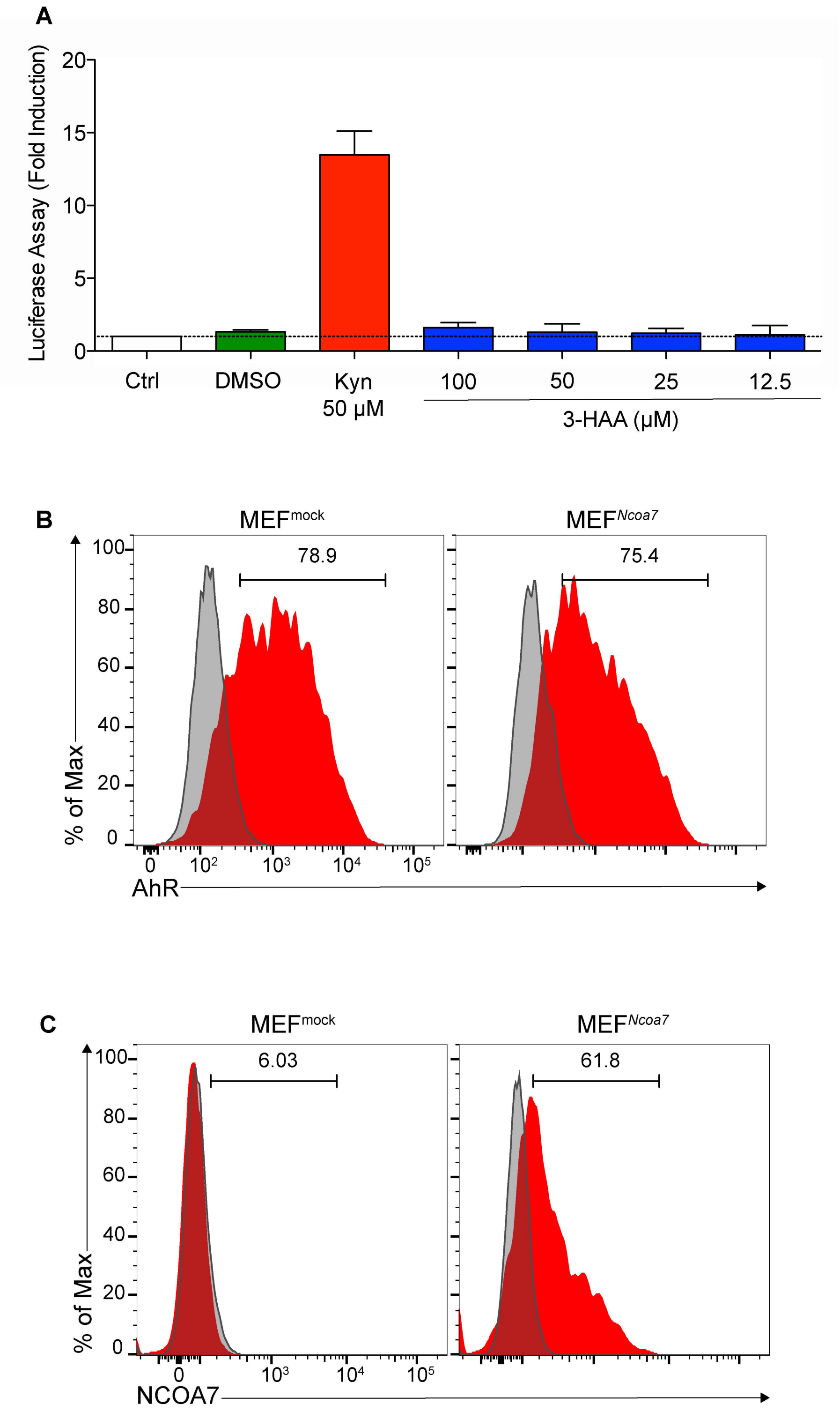

Figure S6 | Lack of AhR activation by 3-HAA alone in H1L1 cells and AhR and NCOA7 expressions in MEFs. **(A)** Transactivation activity of AhR by Kyn or 3-HAA at different concentrations in H1L1 cells. Expression of AhR (**B**) and NCOA7 (**C**) was evaluated by cytofluorometric analysis in MEFs, either mock-transfected (control) or transfected with an NCOA7-encoding vector. A rabbit IgG and a rat IgG_{2a} were used as isotype controls for NCOA7 and AhR, respectively (grey histogram). One representative experiment of two.

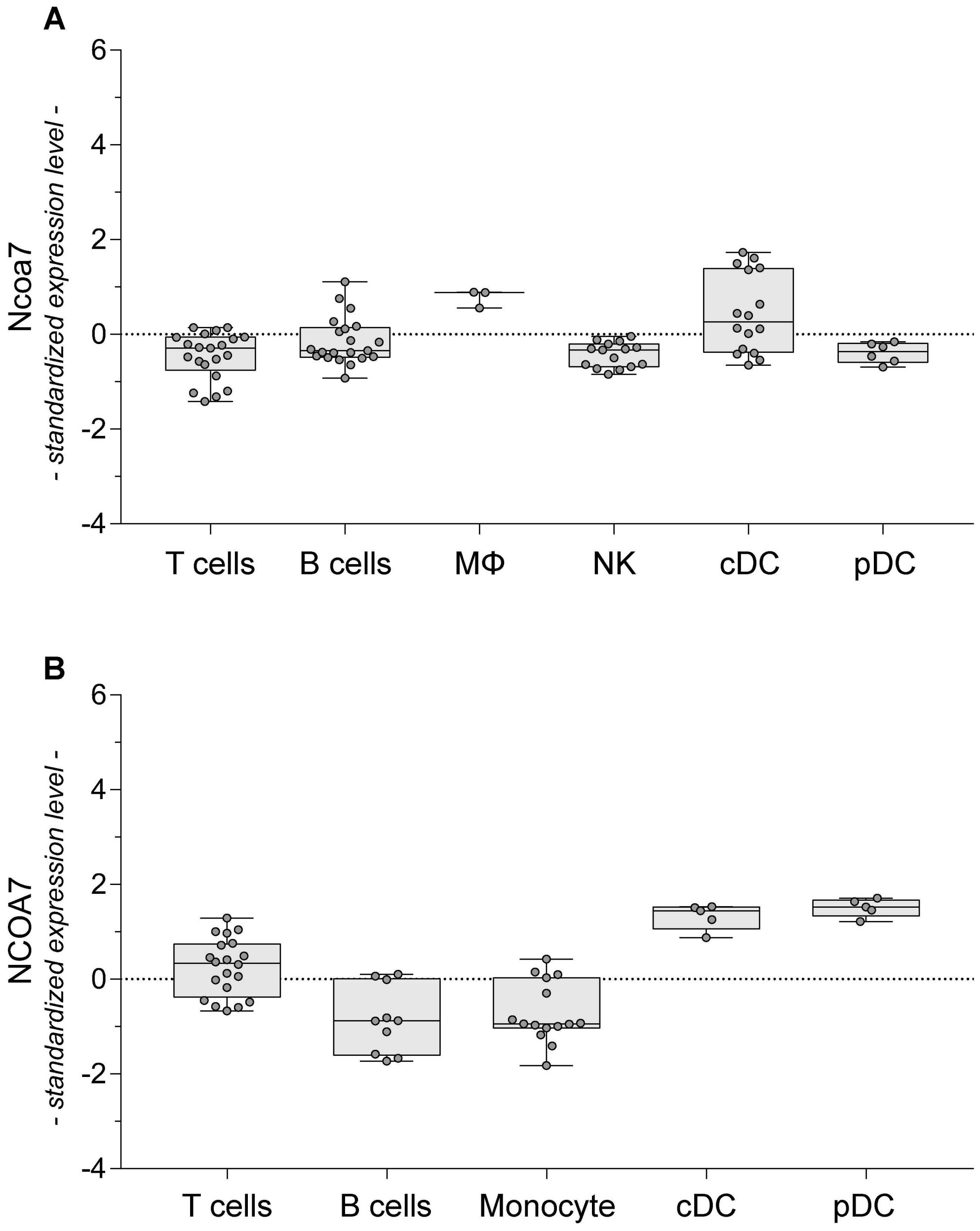

Figure S7 | Expression of *Ncoa7* (A) and *NCOA7* (B) transcript in different types of immune cells from mouse spleens and human peripheral blood mononuclear cells, respectively.

A

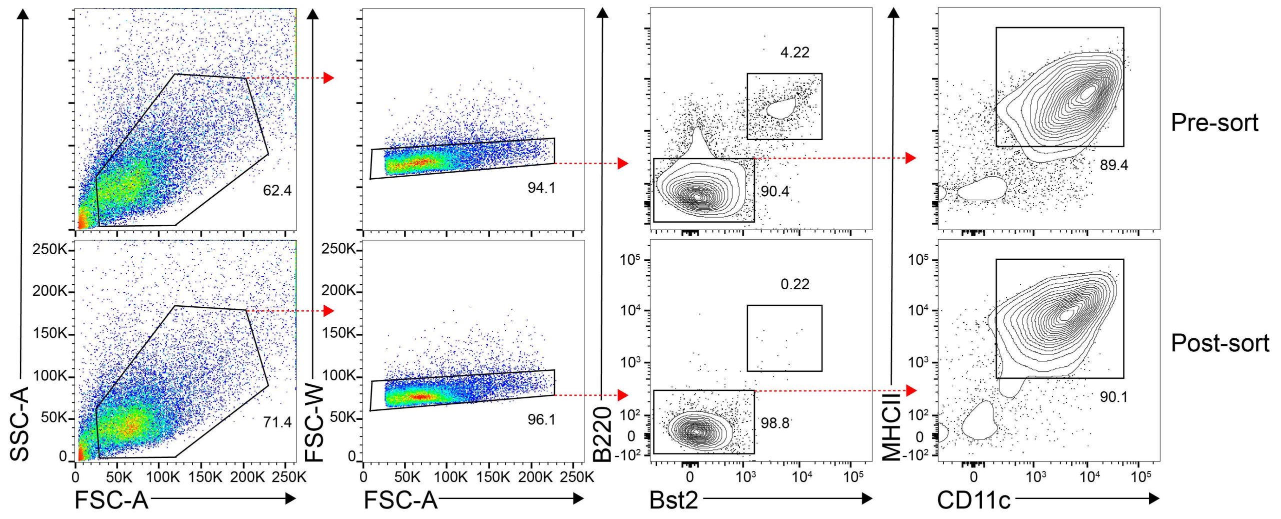

B

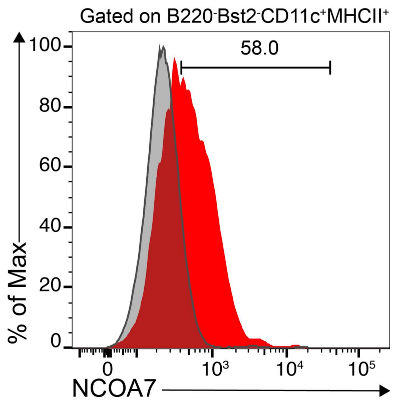

Figure S8| Bone marrow-derived cDCs express NCOA7. (**A**) Bone marrow cells were cultured for nine days with Flt3L and analyzed by FACS to determine the relative percentage of different DC subsets. Shown are the gating strategy to identify pDCs (Bst2$^+$ B220$^+$) and cDCs (CD11c$^+$ MHCII$^+$) and the post-sorting purity of the indicated population. Numbers represent the percentage of cells in the indicated gates. (**B**) NCOA7 expression in gated Bst2$^-$B220$^-$CD11c$^+$MHCII$^+$ cells. NCOA7 expression was evaluated by using an NCOA7-specific antibody, whereas a rabbit IgG was used as isotype control (grey histogram). One representative experiment of two.

Supplementary Table 1. Primer sequences used in this study

Gene	Primer sequence	
β-Actin	F	5'- GGCTCCTAGCACCATGAAGA -3'
	R	5'- AGCTCAGTAACAGTCCGCC-3'
Ncoa7	F	5'- AAA GAC GCC TTG CCA TCT GA -3'
	R	5'- CTC CCC ATT CTG CTG TCG TT -3'

Abbreviations: F, forward; R, reverse.

Supplementary Table 2. Samples of GSE15907 used in this study.

GEO accession	array file name	sample name	cell type	source	source (extended)
GSM399365	GSM399365_EA07068_55678_MoGene_T.4FP3+25+.Sp_#2.cel	T.4FP3+25+.Sp_2	T-cell	spleen	spleen
GSM399366	GSM399366_EA07068_55679_MoGene_T.4FP3+25+.Sp_#3.cel	T.4FP3+25+.Sp_3	T-cell	spleen	spleen
GSM538362	GSM538362_EA07068_85523_MoGene_T.4MEM.LN#1.CEL	T.4Mem.LN_1	T-cell	lymphNode	Lymph-node
GSM538363	GSM538363_EA07068_85524_MoGene_T.4MEM.LN#2.CEL	T.4Mem.LN_2	T-cell	lymphNode	Lymph-node
GSM538364	GSM538364_EA07068_85525_MoGene_T.4MEM.LN#3.CEL	T.4Mem.LN_3	T-cell	lymphNode	Lymph-node
GSM538365	GSM538365_EA07068_58854_MoGene_T.4Mem.Sp_#1.CEL	T.4Mem.Sp_1	T-cell	spleen	spleen
GSM538366	GSM538366_EA07068_58855_MoGene_T.4Mem.Sp_#4.CEL	T.4Mem.Sp_4	T-cell	spleen	spleen
GSM538367	GSM538367_EA07068_58856_MoGene_T.4Mem.Sp_#5.CEL	T.4Mem.Sp_5	T-cell	spleen	spleen
GSM538368	GSM538368_EA07068_96415_MoGene_T.4MEM44H62L.LN#1.CEL	T.4Mem44h62l.LN_1	T-cell	lymphNode	Lymph-node
GSM538369	GSM538369_EA07068_96416_MoGene_T.4MEM44H62L.LN#2.CEL	T.4Mem44h62l.LN_2	T-cell	lymphNode	Lymph-node
GSM538370	GSM538370_EA07068_96417_MoGene_T.4MEM44H62L.LN#3.CEL	T.4Mem44h62l.LN_3	T-cell	lymphNode	Lymph-node
GSM538371	GSM538371_EA07068_96418_MoGene_T.4MEM44H62L.SP#1.CEL	T.4Mem44h62l.Sp_1	T-cell	spleen	spleen
GSM538372	GSM538372_EA07068_96419_MoGene_T.4MEM44H62L.SP#2.CEL	T.4Mem44h62l.Sp_2	T-cell	spleen	spleen
GSM538373	GSM538373_EA07068_96420_MoGene_T.4MEM44H62L.SP#3.CEL	T.4Mem44h62l.Sp_3	T-cell	spleen	spleen
GSM538374	GSM538374_EA07068_52756_MoGene_T.4Nve.LN_#1.CEL	T.4Nve.LN_1	T-cell	lymphNode	Lymph-node
GSM538375	GSM538375_EA07068_52757_MoGene_T.4Nve.LN_#2.CEL	T.4Nve.LN_2	T-cell	lymphNode	Lymph-node
GSM538376	GSM538376_EA07068_52758_MoGene_T.4Nve.LN_#3.CEL	T.4Nve.LN_3	T-cell	lymphNode	Lymph-node
GSM538377	GSM538377_EA07068_55671_MoGene_T.4Nve.MLN_#1.CEL	T.4Nve.MLN_1	T-cell	lymphNode	Mesenteric Lymph-node
GSM538378	GSM538378_EA07068_55672_MoGene_T.4Nve.MLN_#2.CEL	T.4Nve.MLN_2	T-cell	lymphNode	Mesenteric Lymph-node
GSM538379	GSM538379_EA07068_55673_MoGene_T.4Nve.MLN_#3.CEL	T.4Nve.MLN_3	T-cell	lymphNode	Mesenteric Lymph-node
GSM538382	GSM538382_EA07068_108769_MoGene_T.4NVE.SP#5.CEL	T.4Nve.Sp_5	T-cell	spleen	spleen
GSM538383	GSM538383_EA07068_108770_MoGene_T.4NVE.SP#6.CEL	T.4Nve.Sp_6	T-cell	spleen	spleen
GSM538384	GSM538384_EA07068_111386_MoGene_T.4NVE.SP#7.CEL	T.4Nve.Sp_7	T-cell	spleen	spleen
GSM538395	GSM538395_EA07068_85520_MoGene_T.8MEM.LN#1.CEL	T.8Mem.LN_1	T-cell	lymphNode	Lymph-node
GSM538396	GSM538396_EA07068_85521_MoGene_T.8MEM.LN#2.CEL	T.8Mem.LN_2	T-cell	lymphNode	Lymph-node
GSM538397	GSM538397_EA07068_85522_MoGene_T.8MEM.LN#3.CEL	T.8Mem.LN_3	T-cell	lymphNode	Lymph-node
GSM538398	GSM538398_EA07068_58857_MoGene_T.8Mem.Sp_#1.CEL	T.8Mem.Sp_1	T-cell	spleen	spleen
GSM538399	GSM538399_EA07068_58858_MoGene_T.8Mem.Sp_#3.CEL	T.8Mem.Sp_3	T-cell	spleen	spleen
GSM538400	GSM538400_EA07068_58859_MoGene_T.8Mem.Sp_#4.CEL	T.8Mem.Sp_4	T-cell	spleen	spleen
GSM538406	GSM538406_EA07068_52759_MoGene_T.8Nve.LN_#1.CEL	T.8Nve.LN_1	T-cell	lymphNode	Lymph-node
GSM538407	GSM538407_EA07068_52760_MoGene_T.8Nve.LN_#2.CEL	T.8Nve.LN_2	T-cell	lymphNode	Lymph-node
GSM538408	GSM538408_EA07068_52761_MoGene_T.8Nve.LN_#3.CEL	T.8Nve.LN_3	T-cell	lymphNode	Lymph-node
GSM538409	GSM538409_EA07068_58842_MoGene_T.8Nve.MLN_#1.CEL	T.8Nve.MLN_1	T-cell	lymphNode	Mesenteric Lymph-node
GSM538410	GSM538410_EA07068_58843_MoGene_T.8Nve.MLN_#3.CEL	T.8Nve.MLN_3	T-cell	lymphNode	Mesenteric Lymph-node
GSM538411	GSM538411_EA07068_58844_MoGene_T.8Nve.MLN_#4.CEL	T.8Nve.MLN_4	T-cell	lymphNode	Mesenteric Lymph-node
GSM538415	GSM538415_EA07068_52753_MoGene_T.8Nve.Sp_#1.CEL	T.8Nve.Sp_1	T-cell	spleen	spleen
GSM538416	GSM538416_EA07068_52754_MoGene_T.8Nve.Sp_#2.CEL	T.8Nve.Sp_2	T-cell	spleen	spleen
GSM538417	GSM538417_EA07068_52755_MoGene_T.8Nve.Sp_#3.CEL	T.8Nve.Sp_3	T-cell	spleen	spleen
GSM605766	GSM605766_EA07068_55683_MoGene_T.4FP3-.Sp_#1.CEL	T.4FP3-.Sp_1	T-cell	spleen	spleen
GSM605767	GSM605767_EA07068_55684_MoGene_T.4FP3-.Sp_#2.CEL	T.4FP3-.Sp_2	T-cell	spleen	spleen
GSM605768	GSM605768_EA07068_55685_MoGene_T.4FP3-.Sp_#3.CEL	T.4FP3-.Sp_3	T-cell	spleen	spleen
GSM605769	GSM605769_EA07068_111387_MoGene_T.4NVE.SP#8.CEL	T.4Nve.Sp_8	T-cell	spleen	spleen
GSM538201	GSM538201_EA07068_80058_MoGene_B6SPLFO#1.CEL	B.Fo.Sp_1	B-cell	spleen	spleen
GSM538202	GSM538202_EA07068_80059_MoGene_B6SPLFO#2.CEL	B.Fo.Sp_2	B-cell	spleen	spleen
GSM538203	GSM538203_EA07068_80060_MoGene_B6SPLFO#3.CEL	B.Fo.Sp_3	B-cell	spleen	spleen
GSM538207	GSM538207_EA07068_80057_MoGene_B.GC.SP#1.CEL	B.GC.Sp_1	B-cell	spleen	spleen
GSM538208	GSM538208_EA07068_81307_MoGene_B.GC.SP#2.CEL	B.GC.Sp_2	B-cell	spleen	spleen
GSM538209	GSM538209_EA07068_81308_MoGene_B.GC.SP#3.CEL	B.GC.Sp_3	B-cell	spleen	spleen
GSM538213	GSM538213_EA07068_56630_MoGene_B.T1.Sp_#1.CEL	B.T1.Sp_1	B-cell	spleen	spleen
GSM538214	GSM538214_EA07068_56631_MoGene_B.T1.Sp_#2.CEL	B.T1.Sp_2	B-cell	spleen	spleen
GSM538215	GSM538215_EA07068_56632_MoGene_B.T1.Sp_#3.CEL	B.T1.Sp_3	B-cell	spleen	spleen
GSM538216	GSM538216_EA07068_56633_MoGene_B.T2.Sp_#1.CEL	B.T2.Sp_1	B-cell	spleen	spleen
GSM538217	GSM538217_EA07068_56634_MoGene_B.T2.Sp_#2.CEL	B.T2.Sp_2	B-cell	spleen	spleen
GSM538218	GSM538218_EA07068_56635_MoGene_B.T2.Sp_#3.CEL	B.T2.Sp_3	B-cell	spleen	spleen
GSM538219	GSM538219_EA07068_56636_MoGene_B.T3.Sp_#1.CEL	B.T3.Sp_1	B-cell	spleen	spleen
GSM538220	GSM538220_EA07068_56637_MoGene_B.T3.Sp_#2.CEL	B.T3.Sp_2	B-cell	spleen	spleen
GSM538221	GSM538221_EA07068_56638_MoGene_B.T3.Sp_#3.CEL	B.T3.Sp_3	B-cell	spleen	spleen
GSM538225	GSM538225_EA07068_56624_MoGene_B1a.Sp_#1.CEL	B1a.Sp_1	B-cell	spleen	spleen
GSM538226	GSM538226_EA07068_56625_MoGene_B1a.Sp_#2.CEL	B1a.Sp_2	B-cell	spleen	spleen
GSM538227	GSM538227_EA07068_56626_MoGene_B1a.Sp_#3.CEL	B1a.Sp_3	B-cell	spleen	spleen
GSM777019	GSM777019_EA07068_124592_MOGENE-1_0-ST-V1_B.FO.LN_1.CEL	B.Fo.LN_1	B-cell	lymphNode	Lymph-node
GSM777020	GSM777020_EA07068_124593_MOGENE-1_0-ST-V1_B.FO.LN_2.CEL	B.Fo.LN_2	B-cell	lymphNode	Lymph-node
GSM777021	GSM777021_EA07068_124589_MOGENE-1_0-ST-V1_B.FO.MLN_1.CEL	B.Fo.MLN_1	B-cell	lymphNode	Mesenteric Lymph-node
GSM777022	GSM777022_EA07068_124590_MOGENE-1_0-ST-V1_B.FO.MLN_2.CEL	B.Fo.MLN_2	B-cell	lymphNode	Mesenteric Lymph-node
GSM777023	GSM777023_EA07068_124591_MOGENE-1_0-ST-V1_B.FO.MLN_3.CEL	B.Fo.MLN_3	B-cell	lymphNode	Mesenteric Lymph-node
GSM854229	GSM854229_EA07068_105230_MoGene_B1A.SP_1.CEL	B1a.Sp_4	B-cell	spleen	spleen
GSM854230	GSM854230_EA07068_105231_MoGene_B1A.SP_2.CEL	B1a.Sp_5	B-cell	spleen	spleen
GSM854231	GSM854231_EA07068_105232_MoGene_B1A.SP_3.CEL	B1a.Sp_6	B-cell	spleen	spleen
GSM605853	GSM605853_EA07068_105221_MoGene_MF.RP.SP#1.CEL	MF.RP.Sp_1	Macrophage	spleen	spleen
GSM605854	GSM605854_EA07068_105222_MoGene_MF.RP.SP#2.CEL	MF.RP.Sp_2	Macrophage	spleen	spleen
GSM605855	GSM605855_EA07068_105223_MoGene_MF.RP.SP#3.CEL	MF.RP.Sp_3	Macrophage	spleen	spleen
GSM854315	GSM854315_EA07068_140214_MOGENE-1_0-ST-V1_MF.169+11CHI.SLN_1.CEL	MF.169+11chi.SLN_1	Macrophage	lymphNode	Skin-draining Lymph-node
GSM854316	GSM854316_EA07068_140215_MOGENE-1_0-ST-V1_MF.169+11CHI.SLN_2.CEL	MF.169+11chi.SLN_2	Macrophage	lymphNode	Skin-draining Lymph-node
GSM854322	GSM854322_EA07068_140211_MOGENE-1_0-ST-V1_MF.MEDL.SLN_1.CEL	MF.Medl.SLN_1	Macrophage	lymphNode	Skin-draining Lymph-node
GSM854323	GSM854323_EA07068_140212_MOGENE-1_0-ST-V1_MF.MEDL.SLN_2.CEL	MF.Medl.SLN_2	Macrophage	lymphNode	Skin-draining Lymph-node
GSM854324	GSM854324_EA07068_140209_MOGENE-1_0-ST-V1_MF.SBCAPS.SLN_2.CEL	MF.Sbcaps.SLN_2	Macrophage	lymphNode	Skin-draining Lymph-node
GSM854325	GSM854325_EA07068_140210_MOGENE-1_0-ST-V1_MF.SBCAPS.SLN_3.CEL	MF.Sbcaps.SLN_3	Macrophage	lymphNode	Skin-draining Lymph-node
GSM538285	GSM538285_EA07068_96472_MoGene_NK.49CI+.SP#1@N2.CEL	NK.49CI+.Sp_1	Nk	spleen	spleen

GEO accession	array file name	sample name	cell type	source	source (extended)
GSM538286	GSM538286_EA07068_96473_MoGene_NK.49CI+.SP#2.CEL	NK.49CI+.Sp_2	Nk	spleen	spleen
GSM538287	GSM538287_EA07068_96474_MoGene_NK.49CI+.SP#3.CEL	NK.49CI+.Sp_3	Nk	spleen	spleen
GSM538288	GSM538288_EA07068_96475_MoGene_NK.49CI-.SP#1@N2.CEL	NK.49CI-.Sp_1	Nk	spleen	spleen
GSM538289	GSM538289_EA07068_96476_MoGene_NK.49CI-.SP#2.CEL	NK.49CI-.Sp_2	Nk	spleen	spleen
GSM538290	GSM538290_EA07068_96477_MoGene_NK.49CI-.SP#3.CEL	NK.49CI-.Sp_3	Nk	spleen	spleen
GSM538315	GSM538315_EA07068_86161_MoGene_NK.SP#7.CEL	NK.Sp_7	Nk	spleen	spleen
GSM538316	GSM538316_EA07068_86162_MoGene_NK.SP#8.CEL	NK.Sp_8	Nk	spleen	spleen
GSM538317	GSM538317_EA07068_86163_MoGene_NK.SP#9.CEL	NK.Sp_9	Nk	spleen	spleen
GSM605814	GSM605814_EA07068_108027_MoGene_NK.49H+.SP#1.CEL	NK.49H+.Sp_4	Nk	spleen	spleen
GSM605815	GSM605815_EA07068_108028_MoGene_NK.49H+.SP#2.CEL	NK.49H+.Sp_5	Nk	spleen	spleen
GSM605816	GSM605816_EA07068_108029_MoGene_NK.49H+.SP#3.CEL	NK.49H+.Sp_6	Nk	spleen	spleen
GSM605817	GSM605817_EA07068_108030_MoGene_NK.49H-.SP#1.CEL	NK.49H-.Sp_4	Nk	spleen	spleen
GSM605818	GSM605818_EA07068_108031_MoGene_NK.49H-.SP#2.CEL	NK.49H-.Sp_5	Nk	spleen	spleen
GSM605819	GSM605819_EA07068_108032_MoGene_NK.49H-.SP#3.CEL	NK.49H-.Sp_6	Nk	spleen	spleen
GSM538242	GSM538242_EA07068_91083_MoGene_DC2.MLN#1.CEL	DC.4+.MLN_1	cDC	lymphNode	Mesenteric LN
GSM538243	GSM538243_EA07068_91084_MoGene_DC2.MLN#2.CEL	DC.4+.MLN_2	cDC	lymphNode	Mesenteric LN
GSM538244	GSM538244_EA07068_91085_MoGene_DC2.MLN#3.CEL	DC.4+.MLN_3	cDC	lymphNode	Mesenteric LN
GSM538245	GSM538245_EA07068_91077_MoGene_DC2.LN#1.CEL	DC.4+.SLN_1	cDC	lymphNode	Subcutaneous LNs
GSM538246	GSM538246_EA07068_91078_MoGene_DC2.LN#2.CEL	DC.4+.SLN_2	cDC	lymphNode	Subcutaneous LNs
GSM538247	GSM538247_EA07068_91079_MoGene_DC2.LN#3.CEL	DC.4+.SLN_3	cDC	lymphNode	Subcutaneous LNs
GSM538248	GSM538248_EA07068_87573_MoGene_DC2.SP#5.CEL	DC.4+.Sp.ST_5	cDC	spleen	spleen
GSM538249	GSM538249_EA07068_87575_MoGene_DC2.SP#7.CEL	DC.4+.Sp.ST_7	cDC	spleen	spleen
GSM538250	GSM538250_EA07068_90311_MoGene_DC2.SP#8.CEL	DC.4+.Sp.ST_8	cDC	spleen	spleen
GSM538251	GSM538251_EA07068_90312_MoGene_DC2.SP#9.CEL	DC.4+.Sp.ST_9	cDC	spleen	spleen
GSM538252	GSM538252_EA07068_91080_MoGene_DC1.MLN#1.CEL	DC.8+.MLN_1	cDC	lymphNode	Mesenteric LN
GSM538253	GSM538253_EA07068_91081_MoGene_DC1.MLN#2.CEL	DC.8+.MLN_2	cDC	lymphNode	Mesenteric LN
GSM538254	GSM538254_EA07068_91082_MoGene_DC1.MLN#3.CEL	DC.8+.MLN_3	cDC	lymphNode	Mesenteric LN
GSM538255	GSM538255_EA07068_91074_MoGene_DC1.LN#1.CEL	DC.8+.SLN_1	cDC	lymphNode	Subcutaneous LNs
GSM538256	GSM538256_EA07068_91075_MoGene_DC1.LN#2.CEL	DC.8+.SLN_2	cDC	lymphNode	Subcutaneous LNs
GSM538257	GSM538257_EA07068_91076_MoGene_DC1.LN#3.CEL	DC.8+.SLN_3	cDC	lymphNode	Subcutaneous LNs
GSM538258	GSM538258_EA07068_87571_MoGene_DC1.SP#5.CEL	DC.8+.Sp.ST_5	cDC	spleen	spleen
GSM538259	GSM538259_EA07068_87572_MoGene_DC1.SP#6.CEL	DC.8+.Sp.ST_6	cDC	spleen	spleen
GSM538260	GSM538260_EA07068_90308_MoGene_DC1.SP#8.CEL	DC.8+.Sp.ST_8	cDC	spleen	spleen
GSM538261	GSM538261_EA07068_90309_MoGene_DC1.SP#9.CEL	DC.8+.Sp.ST_9	cDC	spleen	spleen
GSM538262	GSM538262_EA07068_91087_MoGene_DC3.MLN#3.CEL	DC.8-4-11b+.MLN_3	cDC	lymphNode	Mesenteric LN
GSM538263	GSM538263_EA07068_96434_MoGene_DC.8-4-11B+.MLN#4.CEL	DC.8-4-11b+.MLN_4	cDC	lymphNode	Mesenteric LN
GSM538264	GSM538264_EA07068_96435_MoGene_DC.8-4-11B+.MLN#5.CEL	DC.8-4-11b+.MLN_5	cDC	lymphNode	Mesenteric LN
GSM538265	GSM538265_EA07068_87567_MoGene_DC3.SP#1.CEL	DC.8-4-11b+.Sp_1	cDC	spleen	spleen
GSM538266	GSM538266_EA07068_87568_MoGene_DC3.SP#2.CEL	DC.8-4-11b+.Sp_2	cDC	spleen	spleen
GSM538267	GSM538267_EA07068_87569_MoGene_DC3.SP#3.CEL	DC.8-4-11b+.Sp_3	cDC	spleen	spleen
GSM605826	GSM605826_EA07068_90310_MoGene_DC2.SP#4.CEL	DC.4+.Sp.ST_4	cDC	spleen	spleen
GSM605827	GSM605827_EA07068_90307_MoGene_DC1.SP#7.CEL	DC.8+.Sp.ST_7	cDC	spleen	spleen
GSM605834	GSM605834_EA07068_91088_MoGene_DC4.MLN#1.CEL	DC.8-4-11b-.MLN_1	cDC	lymphNode	mesenteric lymph node
GSM605835	GSM605835_EA07068_91090_MoGene_DC4.MLN#3.CEL	DC.8-4-11b-.MLN_3	cDC	lymphNode	mesenteric lymph node
GSM605836	GSM605836_EA07068_96401_MoGene_DC.8-4-11B-.MLN#6@N2.CEL	DC.8-4-11b-.MLN_6	cDC	lymphNode	mesenteric LNs
GSM605837	GSM605837_EA07068_96395_MoGene_DC.8-4-11B-.SP#1@N2.CEL	DC.8-4-11b-.Sp_1	cDC	spleen	spleen
GSM605838	GSM605838_EA07068_96396_MoGene_DC.8-4-11B-.SP#2@N2.CEL	DC.8-4-11b-.Sp_2	cDC	spleen	spleen
GSM605839	GSM605839_EA07068_110704_MoGene_DC.8-4-11B-.SP#4.CEL	DC.8-4-11b-.Sp_4	cDC	spleen	spleen
GSM854285	GSM854285_EA07068_96431_MoGene_DC.8-4-11B+.SLN_2.CEL	DC.8-4-11b+.SLN_2	cDC	lymphNode	subcutaneous LN
GSM854286	GSM854286_EA07068_96432_MoGene_DC.8-4-11B+.SLN_3.CEL	DC.8-4-11b+.SLN_3	cDC	lymphNode	subcutaneous LN
GSM854287	GSM854287_EA07068_110703_MoGene_DC.8-4-11B+.SLN_4.CEL	DC.8-4-11b+.SLN_4	cDC	lymphNode	subcutaneous LN
GSM854291	GSM854291_EA07068_110700_MoGene_DC.8-4-11B-.SLN_3.CEL	DC.8-4-11b-.SLN_3	cDC	lymphNode	subcutaneous LN
GSM854292	GSM854292_EA07068_110701_MoGene_DC.8-4-11B-.SLN_4.CEL	DC.8-4-11b-.SLN_4	cDC	lymphNode	subcutaneous LN
GSM854293	GSM854293_EA07068_110702_MoGene_DC.8-4-11B-.SLN_5.CEL	DC.8-4-11b-.SLN_5	cDC	lymphNode	subcutaneous LN
GSM605840	GSM605840_EA07068_105309_MoGene_DC.PDC.8+.SP#1.CEL	DC.pDC.8+.Sp_1	pDC	spleen	spleen
GSM605841	GSM605841_EA07068_105310_MoGene_DC.PDC.8+.SP#2.CEL	DC.pDC.8+.Sp_2	pDC	spleen	spleen
GSM605842	GSM605842_EA07068_105311_MoGene_DC.PDC.8+.SP#3.CEL	DC.pDC.8+.Sp_3	pDC	spleen	spleen
GSM605843	GSM605843_EA07068_105312_MoGene_DC.PDC.8-.SP#1.CEL	DC.pDC.8-.Sp_1	pDC	spleen	spleen
GSM605844	GSM605844_EA07068_105313_MoGene_DC.PDC.8-.SP#2.CEL	DC.pDC.8-.Sp_2	pDC	spleen	spleen
GSM605845	GSM605845_EA07068_105314_MoGene_DC.PDC.8-.SP#3.CEL	DC.pDC.8-.Sp_3	pDC	spleen	spleen
GSM854297	GSM854297_EA07068_130451_MOGENE-1_0-ST-V1_DC.PDC.8+.MLN_1.CEL	DC.pDC.8+.MLN_1	pDC	lymphNode	Mesenteric LN
GSM854298	GSM854298_EA07068_130452_MOGENE-1_0-ST-V1_DC.PDC.8+.MLN_2.CEL	DC.pDC.8+.MLN_2	pDC	lymphNode	Mesenteric LN
GSM854299	GSM854299_EA07068_130454_MOGENE-1_0-ST-V1_DC.PDC.8+.SLN_1.CEL	DC.pDC.8+.SLN_1	pDC	lymphNode	Skin LN
GSM854300	GSM854300_EA07068_130455_MOGENE-1_0-ST-V1_DC.PDC.8+.SLN_2.CEL	DC.pDC.8+.SLN_2	pDC	lymphNode	Skin LN
GSM854301	GSM854301_EA07068_130456_MOGENE-1_0-ST-V1_DC.PDC.8+.SLN_3.CEL	DC.pDC.8+.SLN_3	pDC	lymphNode	Skin LN

Supplementary Table 3. Samples of GSE28490 and GSE28491 used in this study.

GEO accession	array file name	sample name	cell type
GSM705302	GSM705302_mRNA_CD4_EXP2.CEL	CD4+ T cells rep1 mRNA (Roche)	T-cell
GSM705303	GSM705303_mRNA_CD4_EXP3.CEL	CD4+ T cells rep2 mRNA (Roche)	T-cell
GSM705304	GSM705304_mRNA_CD4_EXP4.CEL	CD4+ T cells rep3 mRNA (Roche)	T-cell
GSM705305	GSM705305_mRNA_CD4_EXP7.CEL	CD4+ T cells rep4 mRNA (Roche)	T-cell
GSM705306	GSM705306_mRNA_CD4_EXP8.CEL	CD4+ T cells rep5 mRNA (Roche)	T-cell
GSM705312	GSM705312_mRNA_CD8_EXP10.CEL	CD8+ T cells rep1 mRNA (Roche)	T-cell
GSM705313	GSM705313_mRNA_CD8_EXP11.CEL	CD8+ T cells rep2 mRNA (Roche)	T-cell
GSM705314	GSM705314_mRNA_CD8_EXP12.CEL	CD8+ T cells rep3 mRNA (Roche)	T-cell
GSM705315	GSM705315_mRNA_CD8_EXP3.CEL	CD8+ T cells rep4 mRNA (Roche)	T-cell
GSM705316	GSM705316_mRNA_CD8_EXP4.CEL	CD8+ T cells rep5 mRNA (Roche)	T-cell
GSM705412	GSM705412_CD4_16_04_10.CEL	CD4+ T cells rep1 mRNA (HUG)	T-cell
GSM705413	GSM705413_CD4_21_07_10.CEL	CD4+ T cells rep2 mRNA (HUG)	T-cell
GSM705414	GSM705414_CD4_23_04_10.CEL	CD4+ T cells rep3 mRNA (HUG)	T-cell
GSM705415	GSM705415_CD4_23_07_10.CEL	CD4+ T cells rep4 mRNA (HUG)	T-cell
GSM705416	GSM705416_CD4_30_04_10.CEL	CD4+ T cells rep5 mRNA (HUG)	T-cell
GSM705417	GSM705417_CD8_03_08_10.CEL	CD8+ T cells rep1 mRNA (HUG)	T-cell
GSM705418	GSM705418_CD8_05_08_10.CEL	CD8+ T cells rep2 mRNA (HUG)	T-cell
GSM705419	GSM705419_CD8_06_07_10.CEL	CD8+ T cells rep3 mRNA (HUG)	T-cell
GSM705420	GSM705420_CD8_16_07_10.CEL	CD8+ T cells rep4 mRNA (HUG)	T-cell
GSM705421	GSM705421_CD8_30_04_10.CEL	CD8+ T cells rep5 mRNA (HUG)	T-cell
GSM705297	GSM705297_mRNA_CD19_EXP11.CEL	B cells rep1 mRNA (Roche)	B-cell
GSM705298	GSM705298_mRNA_CD19_EXP6.CEL	B cells rep2 mRNA (Roche)	B-cell
GSM705299	GSM705299_mRNA_CD19_EXP7.CEL	B cells rep3 mRNA (Roche)	B-cell
GSM705300	GSM705300_mRNA_CD19_EXP8.CEL	B cells rep4 mRNA (Roche)	B-cell
GSM705301	GSM705301_mRNA_CD19_EXP9.CEL	B cells rep5 mRNA (Roche)	B-cell
GSM705402	GSM705402_B_03_08_10.CEL	B cells rep1 mRNA (HUG)	B-cell
GSM705403	GSM705403_B_05_08_10.CEL	B cells rep2 mRNA (HUG)	B-cell
GSM705404	GSM705404_B_07_07_10.CEL	B cells rep3 mRNA (HUG)	B-cell
GSM705405	GSM705405_B_23_07_10.CEL	B cells rep4 mRNA (HUG)	B-cell
GSM705406	GSM705406_B_27_07_10.CEL	B cells rep5 mRNA (HUG)	B-cell
GSM705287	GSM705287_mRNA_CD14_EXP1.CEL	Monocytes rep1 mRNA (Roche)	Monocyte
GSM705288	GSM705288_mRNA_CD14_EXP13.CEL	Monocytes rep2 mRNA (Roche)	Monocyte
GSM705289	GSM705289_mRNA_CD14_EXP15.CEL	Monocytes rep3 mRNA (Roche)	Monocyte
GSM705290	GSM705290_mRNA_CD14_EXP16.CEL	Monocytes rep4 mRNA (Roche)	Monocyte
GSM705291	GSM705291_mRNA_CD14_EXP17.CEL	Monocytes rep5 mRNA (Roche)	Monocyte
GSM705292	GSM705292_mRNA_CD14_EXP18.CEL	Monocytes rep6 mRNA (Roche)	Monocyte
GSM705293	GSM705293_mRNA_CD14_EXP2.CEL	Monocytes rep7 mRNA (Roche)	Monocyte
GSM705294	GSM705294_mRNA_CD14_EXP6.CEL	Monocytes rep8 mRNA (Roche)	Monocyte
GSM705295	GSM705295_mRNA_CD14_EXP7.CEL	Monocytes rep9 mRNA (Roche)	Monocyte
GSM705296	GSM705296_mRNA_CD14_EXP8.CEL	Monocytes rep10 mRNA (Roche)	Monocyte
GSM705407	GSM705407_CD14_05_08_10.CEL	Monocytes rep1 mRNA (HUG)	Monocyte
GSM705408	GSM705408_CD14_13_07_10.CEL	Monocytes rep2 mRNA (HUG)	Monocyte
GSM705409	GSM705409_CD14_23_04_10.CEL	Monocytes rep3 mRNA (HUG)	Monocyte
GSM705410	GSM705410_CD14_27_08_10.CEL	Monocytes rep4 mRNA (HUG)	Monocyte
GSM705411	GSM705411_CD14_30_07_10.CEL	Monocytes rep5 mRNA (HUG)	Monocyte

GSM705321	GSM705321_mRNA_mDC_EXP14.CEL	mDC rep1 mRNA (Roche)	cDC
GSM705322	GSM705322_mRNA_mDC_EXP15.CEL	mDC rep2 mRNA (Roche)	cDC
GSM705323	GSM705323_mRNA_mDC_EXP16.CEL	mDC rep3 mRNA (Roche)	cDC
GSM705324	GSM705324_mRNA_mDC_EXP17.CEL	mDC rep4 mRNA (Roche)	cDC
GSM705325	GSM705325_mRNA_mDC_EXP18.CEL	mDC rep5 mRNA (Roche)	cDC
GSM705329	GSM705329_mRNA_pDC_EXP13.CEL	pDC rep1 mRNA (Roche)	pDC
GSM705330	GSM705330_mRNA_pDC_EXP14.CEL	pDC rep2 mRNA (Roche)	pDC
GSM705331	GSM705331_mRNA_pDC_EXP16.CEL	pDC rep3 mRNA (Roche)	pDC
GSM705332	GSM705332_mRNA_pDC_EXP17.CEL	pDC rep4 mRNA (Roche)	pDC
GSM705333	GSM705333_mRNA_pDC_EXP18.CEL	pDC rep5 mRNA (Roche)	pDC

Supplementary Methods

Chemistry of Generation of Biotin−Tagged 3-HAA

The preparation of derivative **1** (Biot1) was accomplished as shown in Scheme 1. 3-hydroxy-2-nitrobenzoic acid was reacted with an equimolar amount of 2-(6-bromohexyl)-1*H*-isoindole-1,3(2*H*)-dione **4** (1), to give the ester **5**, which was successively treated with hydrazine in EtOH at reflux to de-protect the amino group. The reduction of **6** afforded the 2-aminohydroxybenzoate **7**, which was finally conjugated with properly functionalized biotin molecule **8** (2) to give the target derivative **1**. Following an analogous procedure, derivative **2** (Biot2) was synthesized as outlined in Scheme 2. The sequential steps included a coupling reaction of 3-hydroxy-2-nitrobenzoic acid with an excess of **4** (1), to give the di-substituted intermediate **9**, followed by catalytic reduction to the aminoderivative **10**, amino group de-protection to compound **11**, and basic hydrolysis to 2-aminobenzoic acid **12,** which was finally conjugated with biotin derivative **8** (2).

Starting from 3-hydroxybenzoic acid, and following the same procedure used to synthesize Biot2, compound **3** (Biot3) was also prepared, through intermediates **13-15**, as outlined in Scheme 3.

Scheme 1[a]

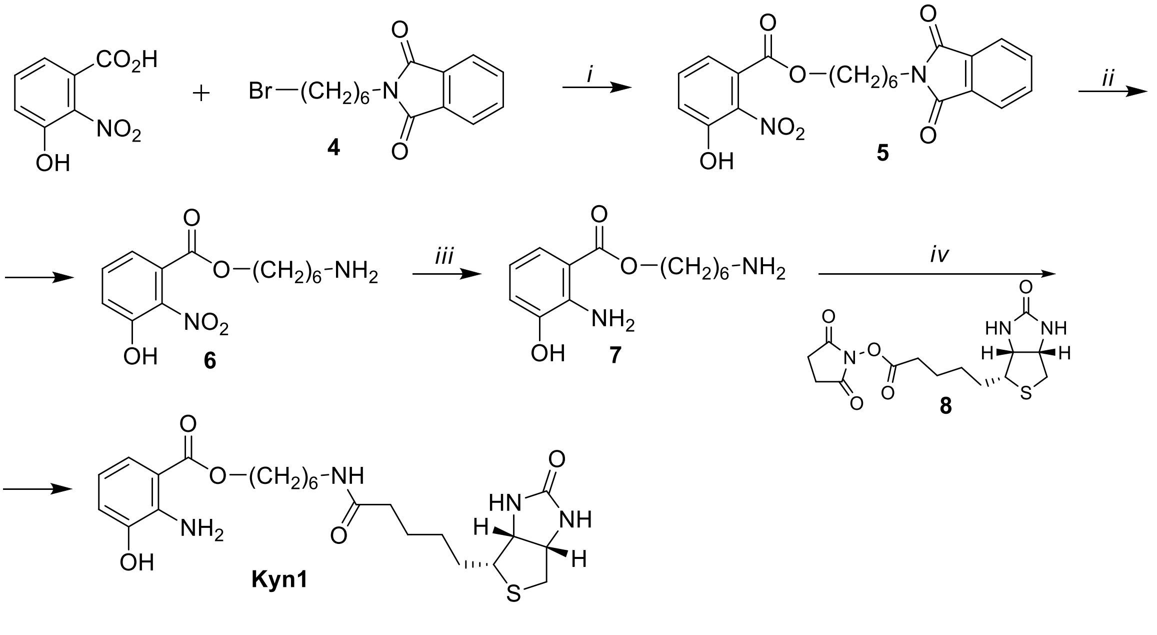

[a] Reagents: (i) Cs_2CO_3, DMF, 70 °C; (ii) $H_2N\text{-}NH_2 \cdot H_2O$, EtOH, reflux; (iii) H_2, Raney Ni, EtOH/DMF; (iv) DMF.

Scheme 2[a]

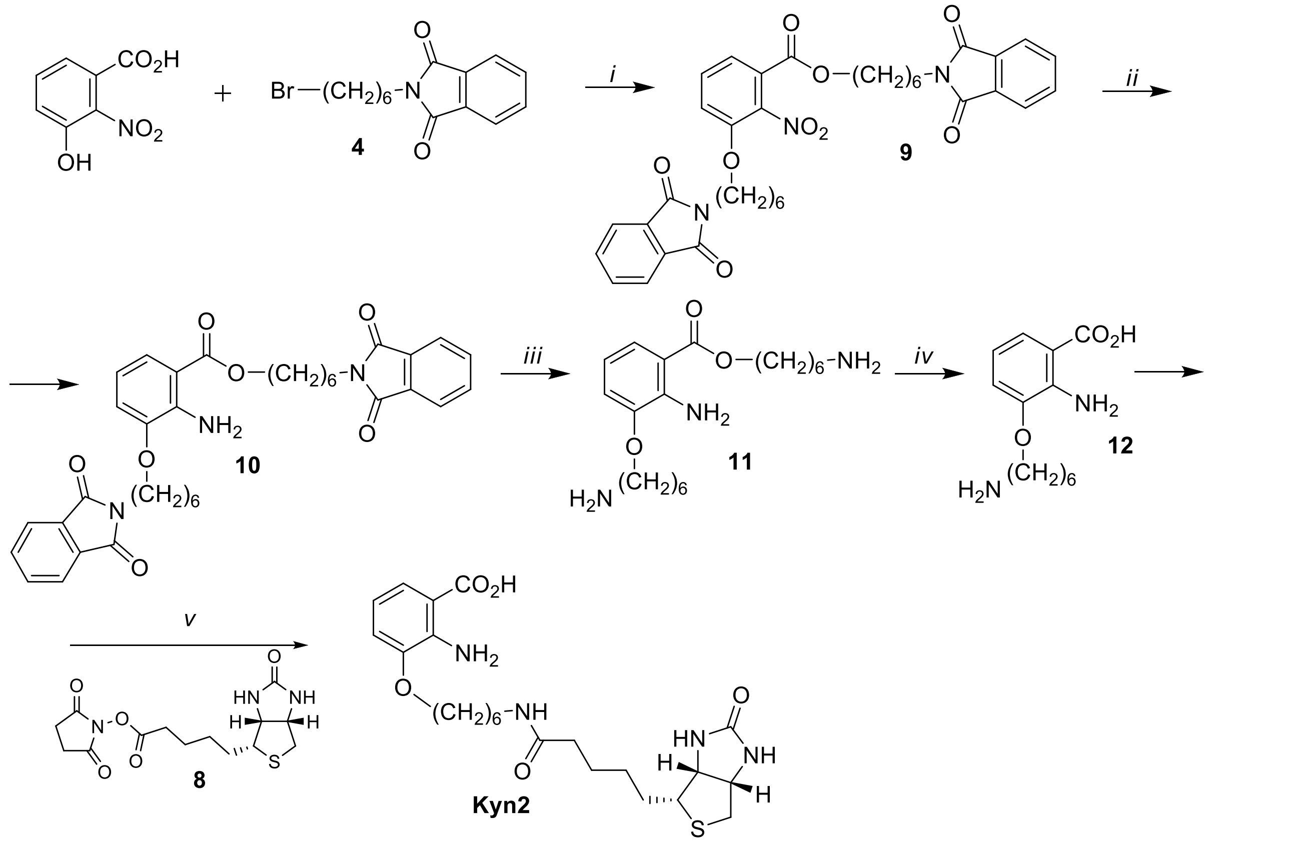

[a] Reagents: (i) Cs_2CO_3, DMF, 70 °C; (ii) H_2, Raney Ni, EtOH/DMF; (iii) $H_2N\text{-}NH_2 \cdot H_2O$, EtOH, reflux; (iv) 4% NaOH, reflux; (v) DMF.

Scheme 3[a]

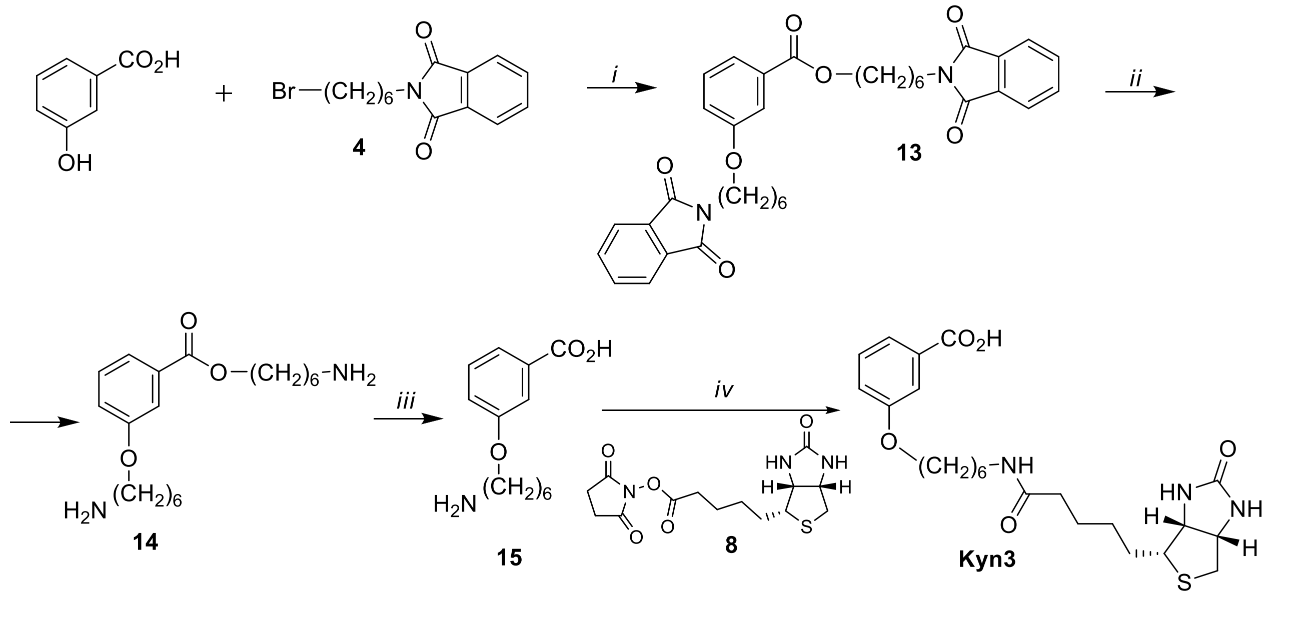

[a] Reagents: (i) Cs_2CO_3, DMF, 70 °C; (ii) $H_2N\text{-}NH_2 \cdot H_2O$, EtOH, reflux; (iii) 4% NaOH, reflux; (iv) DMF.

Experimental Section

6-(1,3-Dioxo-1,3-dihydro-2*H*-isoindol-2-yl)hexyl 3-hydroxy-2-nitrobenzoate (5). A mixture of 3-hydroxy-2-nitrobenzoic acid (0.30 g, 1.63 mmol), 2-(6-bromohexyl)-1*H*-isoindole-1,3(2*H*)-dione **4** (1) (0.50 g, 1.63 mmol), and Cs_2CO_3 (1.06 g, 3.27 mmol) in DMF (10 mL) was heated at 70 °C. After 4 h, the reaction mixture was poured into ice/water, acidified (pH ~ 1) with 2 N HCl, and extracted with EtOAc. The organic layers were evaporated to dryness to give a residue, which was purified by flash chromatography, by eluting with CH_2Cl_2, to give **5** as an oil (0.34 g, 50%); ^{1}H NMR ($CDCl_3$-d_6) δ 1.45-1.65 and 1.70-1.90 (m, each 4H, CH_2), 3.70 (t, J = 6.6 Hz, 2H, CH_2N), 4.35 (t, J = 6.6 Hz, 2H, OCH_2), 7.10 (d, J = 7.8 Hz, 1H, H-4), 7.30 (d, J = 7.8 Hz, 1H, H-6), 7.60 (t, J = 7.8 Hz, 1H, H-5), 7.70-7.80 and 7.85-7.95 (m, each 2H, aromatic CH).

6-Aminohexyl 3-hydroxy-2-nitrobenzoate (6). A mixture of **5** (0.2 g, 0.48 mmol) and hydrazine hydrate (0.07 g, 1.45 mmol) in EtOH (10 mL) was refluxed for 2 h. After cooling, the reaction mixture was filtered and the filtrate was evaporated to dryness to give a residue which was purified by flash chromatography, by eluting with MeOH/$CHCl_3$ (from 20% to 100%), to give **6** (0.06 g, 44%): mp 175-177 °C; ^{1}H NMR (DMSO-d_6) δ 1.30-1.35 and 1.45-1.65 (m, each 4H, CH_2), 2.70 (t, J = 6.9 Hz, 2H, $\mathit{CH_2}NH_2$), 4.10 (t, J = 6.9 Hz, 2H, OCH_2), 6.40 (d, J = 8.0 Hz, 1H, H-4), 7.30 (d, J = 8.0 Hz, 1H, H-6), 7.60 (t, J = 8.0 Hz, 1H, H-5).

6-Aminohexyl 2-amino-3-hydroxybenzoate (7). A stirred solution of **6** (0.38 g, 1.34 mmol) in EtOH/DMF was hydrogenated over a catalytic amount of Raney Nickel at room temperature and atmospheric pressure for 30 min. The mixture was then filtered over Celite, and the filtrate was evaporated to dryness to afford a residue which was purified by flash chromatography, eluting with MeOH/$CHCl_3$ (20%), to give **7** (0.18 g, 53%): mp 138-140 °C; ^{1}H NMR (DMSO-d_6) δ 1.30-1.45 and 1.50-1.70 (m, each 4H, CH_2), 2.85 (t, J = 7.2 Hz, 2H, $\mathit{CH_2}NH_2$), 4.20 (t, J = 7.2 Hz, 2H, OCH_2), 6.10 (bs, 2H, NH_2), 6.40 (t, J = 7.8 Hz, 1H, H-5), 6.80 (d, J = 7.8 Hz, 1H, H-4), 7.20 (d, J = 7.8 Hz, 1H, H-6), 7.90 (bs, 2H, $CH_2\mathit{NH_2}$).

6-({5-[(3a*R*,4*S*,6a*S*)-2-Oxohexahydro-1*H*-thieno[3,4-*d*]imidazol-4-yl]pentanoyl}amino)hexyl 2-amino-3-hydroxybenzoate (Biot1). A mixture of **7** (0.16 g, 0.63 mmol) and **8 (2)** (0.26 g, 0.76 mmol) in DMF (20 mL) was maintained at room temperature for 4 h. The mixture was poured into ice/water and neutralized with a saturated solution of $NaHCO_3$. The obtained precipitate was filtered and purified by flash chromatography, by eluting with MeOH/$CHCl_3$ (2%), to give Biot1 (0.05 g, 16%): mp 88-89 °C; ^{1}H NMR (DMSO-d_6) δ 1.20-1.70 (m, 14H, CH_2), 2.05 (t, J = 7.2 Hz, 2H, CH_2CO), 2.55 (d, J = 12.4 Hz, 1H, H-6'), 2.80 (dd, J = 5.0 and 12.4 Hz, 1H, H-6'), 3.00 (q, J = 6.0 Hz, 1H, CH_2N), 3.10 (m, 1H, H-4'), 4.05-4.15 (m, 1H, H-3a'), 4.15 (t, J = 6.4 Hz, 1H, OCH_2), 4.30 (m, 1H,

H-6a'), 6.15 (bs, 2H, NH_2), 6.35-6.45 (m, 3H, NH and H-5), 6.80 (d, J = 6.8 Hz, 1H, H-4), 7.25 (d, J = 8.0 Hz, 1H, H-6), 7.75 (t, J = 5.4 Hz, 1H, NHCO), 9.80 (s, 1H, OH). ^{13}C NMR (DMSO-d_6) δ 25.74, 25.78, 26.51, 28.48, 28.65, 29.50, 35.66, 38.72, 40.27, 55.88, 59.62, 61.48, 64.16, 109.72, 114.72, 117.21, 121.09, 141.43, 145.02, 163.13, 168.11, 172.24. Anal. Calcd for $C_{23}H_{34}N_4O_5S$: C, 57,72; H, 7.16; N, 11.71. Found: C, 57,94; H, 7.35 N, 11.60.

6-(1,3-Dioxo-1,3-dihydro-2*H*-isoindol-2-yl)hexyl 3-{[6-(1,3-dioxo-1,3-dihydro-2*H*-isoindol-2-yl)hexyl]oxy}-2-nitrobenzoate (9). A mixture of 3-hydroxy-2-nitrobenzoic acid (1.3 g, 7.09 mmol), 2-(6-bromohexyl)-1***H***-isoindole-1,3(2***H***)-dione **4** (1) (5.5 g, 17.7 mmol), and Cs_2CO_3 (5.7 g, 17.7 mmol) in DMF (20 mL) was heated at 70 °C. After 2 h the reaction mixture was poured into ice/water and extracted with EtOAc. The organic layers were evaporated to dryness, to give a residue, which was purified by flash chromatography, eluting with EtOAc/petroleum ether (from 35% to 50%), to give **9** as an oil (0.45 g, 99%); ^{1}H NMR ($CDCl_3$) δ 1.35-1.80 (m, 16H, CH_2), 3.70 (t, J = 7.0 Hz, 4H, CH_2N), 4.10 and 4.30 (t, J = 6.2 Hz, each 2H, OCH_2), 7.20 (d, J = 8.4 Hz, 1H, H-4), 7.45 (t, J = 8.1 Hz, 1H, H-5), 7.60 (d, J = 7.8 Hz, 1H, H-6), 7.65-7.75 and 7.80-7.90 (m, each 4H, aromatic CH).

6-(1,3-Dioxo-1,3-dihydro-2*H*-isoindol-2-yl)hexyl 2-amino-3-{[6-(1,3-dioxo-1,3-dihydro-2*H*-isoindol-2-yl)hexyl]oxy}benzoate (10). Staring from **9** and following the same procedure as employed to synthesize **7**, the title compound was prepared at a 72% yield: mp 98-99 °C; ^{1}H NMR ^{1}H NMR ($CDCl_3$) δ 1.35-1.80 (m, 16H, CH_2), 3.70 (t, J = 6.5 Hz, 4H, CH_2N), 4.00 and 4.25 (t, J = 6.2 Hz, each 2H, OCH_2), 6.00 (bs, 2H, NH_2), 6.55 (t, J = 8.0 Hz, 1H, H-5), 6.85 (d, J = 7.5 Hz, 1H, H-4), 7.45 (d, J = 7.7 Hz, 1H, H-6), 7.65-7.75 and 7.80-7.90 (m, each 4H, aromatic CH).

6-Aminohexyl 2-amino-3-[(6-aminohexyl)oxy]benzoate (11). A mixture of **10** (0.68 g, 1.11 mmol) and hydrazine hydrate (0.33 g, 6.66 mmol) in EtOH (20 mL) was refluxed for 6h. After cooling the reaction mixture was filtered and the filtrate was evaporated to dryness to give a residue which was purified by flash chromatography, eluting with $MeOH/CHCl_3/NH_4OH$ (5/45/50), to give **11** (0.35 g, 90%): mp 278-280 °C; ^{1}H NMR (DMSO-d_6) 1.30-1.40 (m, 12H, CH_2), 1.65-1.75 (m, 4H, CH_2), 2.60 (q, J = 6.4 Hz, 4H, CH_2NH_2), 3.95 and 4.15 (t, J = 6.4 Hz, each 2H, OCH_2), 6.20 (bs, 2H, NH_2), 6.45 (t, J = 8.0 Hz, 1H, H-5), 6.95 (d, J = 7.5 Hz, 1H, H-4), 7.25 (d, J = 8.1 Hz, 1H, H-6).

2-Amino-3-[(6-aminohexyl)oxy]benzoic acid (12). A mixture of **11** (0.32 g, 0.91 mmol) and 4% NaOH (5 mL) was refluxed for 4 h. After cooling, the reaction mixture was neutralized with 2N HCl, obtaining a precipitate that was filtered and washed with water and Et_2O, to give **12** (0.16 g, 70%): mp 198-200 °C; ^{1}H NMR (DMSO-d_6) δ 1.35-1.45 (m, 4H, CH_2), 1.50-1.60 and 1.65-1.75 (m, each 2H, CH_2), 2.75 (t, J = 7.2 Hz, 2H, CH_2NH_2), 3.90 (t, J = 6.0 Hz, 2H, OCH_2), 6.35 (t, J = 7.7 Hz, 1H, H-5), 6.70 (d, J = 7.7 Hz, 1H, H-4), 7.35 (d, J = 7.7 Hz, 1H, H-6).

3-{[6-({5-[(3a*R*,4*S*,6a*S*)-2-Oxohexahydro-1*H*-thieno[3,4-*d*]imidazol-4-yl]pentanoyl}amino)hexyl]oxy}-2-aminobenzoic acid (Biot2). Starting from **12** and following the same procedure employed to synthesize Biot1, the title compound was prepared. The mixture was poured into ice/water, obtaining a precipitate which was filtered and purified by flash chromatography, by eluting with MeOH/$CHCl_3$ (2%), to give Biot2 at a 86% yield: mp 154-156 °C; ^{1}H NMR (DMSO-d_6) δ 1.20-1.70 (m, 14H, CH_2), 2.05 (m, 2H, CH_2CO), 2.55 and 2.80 (m, each 1H, H-6'), 3.00-3.10, (m, 3H, CH_2N and H-4'), 3.95 (m, 1H, OCH_2), 4.15 and 4.30 (bs, each 1H, H-3a'and H-6a'), 6.35-6.45 (m, 3H, NH and H-5), 6.90 (bs, 1H, H-4), 7.30 (bs, 1H, H-6), 7.75 (bs, 1H, NHCO). ^{13}C NMR (DMSO-d_6) δ 25.69, 25.79, 26.62, 28.48, 28.66, 29.10, 29.54, 35.67, 38.70, 40.28, 55.88, 59.61, 61.48, 68.45, 114.22, 114.51, 122.88, 142.22, 146.40, 163.14, 170.23, 172.24. Anal. Calcd for $C_{23}H_{34}N_4O_5S$: C, 57,72; H, 7.16; N, 11.71. Found: C, 57,87; H, 7.25; N, 11.68.

6-(1,3-Dioxo-1,3-dihydro-2*H*-isoindol-2-yl)hexyl 3-{[6-(1,3-dioxo-1,3-dihydro-2*H*-isoindol-2-yl)hexyl]oxy}benzoate (13). Starting from 3-hydroxybenzoic acid and following the same procedure as employed to synthesize **9**, the title compound was prepared (12 h) as an oil in 100% yield; in this case. it was purified by flash chromatography, eluting with CH_2Cl_2; ^{1}H NMR ($CDCl_3$) δ 1.35-1.80 (m, 16H, CH_2), 3.65 (t, J = 7.0 Hz, 4H, CH_2N), 3.95 and 4.30 (t, J = 6.3 Hz, each 2H, OCH_2), 7.05 (d, J = 8.1 Hz, 1H, H-4), 7.30 (t, J = 8.1 Hz, 1H, H-5), 7.50 (s, 1H, H-2), 7.60 (d, J = 7.6 Hz, 1H, H-6), 7.65-7.75 and 7.80-7.90 (m, each 4H, aromatic CH).

6-Aminohexyl 3-[(6-aminohexyl)oxy]benzoate (14). Starting from **13** and following the same procedure as employed to synthesize **11**, the title compound was prepared (2 h); in this case the reaction was worked by adding cycloexane/Et_2O to the cooled reaction mixture, obtaining a precipitate which was filtered. The filtrate was evaporated to dryness, to give **14** as an oil in 100% yield; ^{1}H NMR (DMSO-d_6) 1.20-1.40 (m, 12H, CH_2), 1.65-1.75 (m, 4H, CH_2), 2.50 (m, 4H, *CH_2*NH_2), 3.95 and 4.15 (t, J = 6.4 Hz, each 2H, OCH_2), 7.20 (t, J = 8.0 Hz, 1H, H-4), 7.35-7-55 (m, 3H, H-5, H-2, and H-6).

3-[(6-Aminohexyl)oxy]benzoic acid (15). Staring from **15** and following the same procedure as employed to synthesize **12**, the title compound was prepared (3 h); in this case, after cooling, the reaction was worked by acidification with 2N HCl (pH ~ 4), obtaining a precipitate that was filtered; the filtrate was extracted with EtOAc and evaporated to dryness, obtaining a solid, which was united to the precipitate filtered, to give **15** in 61% yield: mp 196-198 °C; ^{1}H NMR (CD_3OD) δ 1.45-1.55 (m, 4H, CH_2), 1.60-1.70 and 1.75-1.85 (m, each 2H, CH_2), 2.95 (t, J = 7.5 Hz, 2H, *CH_2*NH_2), 4.00 (t, J = 6.3 Hz, 2H, OCH_2), 6.65 (dd, J = 2.3 and 8.1 Hz, 1H, H-4), 6.95 (t, J = 7.8 Hz, H-5), 7.15-7.25 (m, 2H, H-2 and H-6).

3-{[6-({5-[(3a*R*,4*S*,6a*S*)-2-Oxohexahydro-1*H*-thieno[3,4-*d*]imidazol-4-yl]pentanoyl}amino)hexyl]oxy}benzoic acid (Biot3). Staring from **15** and following the same procedure as employed to synthesize **Biot1**, the title compound was prepared (4 h) in 5% yield: mp 159-160 °C; ^{1}H NMR (DMSO-d_6) δ 1.25-1.75 (m, 14H, CH_2), 2.05 (m, 2H, CH_2CO), 2.55 (d, J = 12.4 Hz, 1H, H-6'), 2.80 (dd, J = 5.0 and 12.4 Hz, 1H, H-6'), 3.00 (q, J = 6.4 Hz, 2H, CH_2N), 3.10, (m, 1H, H-4'), 3.95 (t, J = 6.4 Hz, 2H, OCH_2), 4.15 and 4.30 (m, each 1H, H-3a' and H-6a'), 6.35 and 6.45 (s, each 1H, NH), 7.00 (d, J = 6.3 Hz, 1H, H-4), 7.25 (t, J = 7.6 Hz, 1H, H-5), 7.45 (bs, 1H, H-2), 7.50 (d, J = 7.6 Hz, 1H, H-6), 7.80 (t, J = 5.3 Hz, 1H, NHCO). ^{13}C NMR (DMSO-d_6) δ 25.70, 25.80, 26.61, 28.49, 28.67, 29.09, 29.54, 35.66, 38.74, 40.27, 55.89, 59.63, 61.50, 67.78, 115.02, 117.47, 121.85, 129,18, 158.73, 163.17, 169.32, 172.25. Anal. Calc. for $C_{23}H_{33}N_3O_5S$: C, 59,59; H, 7.17; N, 9.06. Found: C, 59,70; H, 7.28; N, 9.00.

2-D Electrophoresis, Matrix-Assisted Laser Desorption/Ionization Time-of-Flight Mass Spectrometry (MALTI-TOF-MS) Analysis, and Database Search

These procedures were carried out by Applied Biomics. 2-D cell lysis buffer (30 mM Tris-HCl, pH 8.8, containing 7 M urea, 2 M thiourea and 4% CHAPS) was added to the immunoprecipitate (IP) samples plus 1% SDS to elute the IP protein complex, shaking 2 h at room temperature, and then pass the 0.2 um spin column to collect the eluted proteins in the solution. Then, eluted proteins were subjected to replace the buffer using 5 kDa MWCO spin column with 2D lysis buffer without SDS. Protein assay was carried out using Bio-Rad protein assay method. After loading the labeled samples into a strip holder, isoelectrofocusing (IEF) electrophoresis was performed by the protocol provided by Amersham BioSciences and run under dark at 20 °C. After IEF, strips were incubated in a fresh made equilibration buffer 1 (50 mM Tris-HCl, pH 8.8, containing 6 M urea, 30% glycerol, 2% SDS, bromophenol blue, and 10 mg/ml DTT) for 15 min with slow shaking. Then, strips were rinsed in a fresh made equilibration buffer 2 (50 mM Tris-HCl, pH 8.8, containing 6 M urea, 30% glycerol, 2% SDS, bromophenol blue, and 45 mg/ml iodoacetamide) for 10 min with slow shaking. Strips were then rinsed once in the SDS-gel running buffer before being transferred into the SDS-Gel (12% SDS-gel prepared using low florescent glass plates) and sealed with 0.5%

(w/v) agarose solution (in SDS-gel running buffer). SDS-gels were run at 15 °C and stopped until the dye front running out of the gels.

Spots of interest were picked up by Ettan Spot Picker (GE Healthcare), based on the in-gel analysis and spot picking design by DeCyder software. Gel spots were washed a few times, and digested in-gel with modified porcine trypsin protease (Trypsin Gold, Promega). The digested tryptic peptides were desalted by Zip-tip C18 (Millipore). Peptides were eluted from the Zip-tip with 0.5 μl of matrix solution (α-cyano-4-hydroxycinnamic acid, 5 mg/ml in 50% acetonitrile, 0.1% trifluoroacetic acid, and 25 mM ammonium bicarbonate) and spotted on the MALDI plate.

MALDI-TOF (MS) and TOF/TOF (tandem MS/MS) were performed on a 5800 mass spectrometer (AB Sciex). MALDI-TOF mass spectra were acquired in reflectron positive ion mode, averaging 2,000 laser shots per spectrum. TOF/TOF tandem MS fragmentation spectra were acquired for each sample, averaging 2,000 laser shots per fragmentation spectrum on each of the 5-10 most abundant ions present in each sample (excluding trypsin autolytic peptides and other known background ions).

Both the resulting peptide mass and the associated fragmentation spectra were submitted to GPS Explorer version 3.5 equipped with MASCOT search engine (Matrix science) to search the database of National Center for Biotechnology Information non-redundant (NCBInr) or Swiss Protein database. Searches were performed without constraining protein molecular weight or isoelectric point, with variable carbamidomethylation of cysteine and oxidation of methionine residues, and with one missed cleavage allowed in the search parameters. Candidates with either protein score C.I.% or Ion C.I.% greater than 95 were considered significant.

References

1. J. Jiang, W. Wang, D. C. Sane and B. Wang: Synthesis of RGD analogs as potential vectors for targeted drug delivery. *Bioorg Chem*, 29(6), 357-79 (2001) doi:10.1006/bioo.2001.1227
2. R. Kottani, R. A. Valiulin and A. G. Kutateladze: Direct screening of solution phase combinatorial libraries encoded with externally sensitized photolabile tags. *Proc Natl Acad Sci U S A*, 103(38), 13917-21 (2006) doi:10.1073/pnas.0606380103
